# A Conserved Regulatory Circuit Controls Large Adhesins in Vibrio cholerae

**DOI:** 10.1128/mBio.02822-19

**Published:** 2019-12-03

**Authors:** Giordan Kitts, Krista M. Giglio, David Zamorano-Sánchez, Jin Hwan Park, Loni Townsley, Richard B. Cooley, Benjamin R. Wucher, Karl E. Klose, Carey D. Nadell, Fitnat H. Yildiz, Holger Sondermann

**Affiliations:** a Department of Microbiology and Environmental Toxicology, University of California, Santa Cruz, Santa Cruz, California, USA; b Department of Molecular Medicine, College of Veterinary Medicine, Cornell University, Ithaca, New York, USA; c Department of Biology, South Texas Center for Emerging Infectious Diseases, University of Texas at San Antonio, San Antonio, Texas, USA; d Department of Biological Sciences, Dartmouth, Hanover, New Hampshire, USA; University of Washington

**Keywords:** *Vibrio cholerae*, adhesins, biofilms, cell signaling, proteases

## Abstract

The dinucleotide second messenger c-di-GMP has emerged as a central regulator of reversible cell attachment during bacterial biofilm formation. A prominent cell adhesion mechanism first identified in pseudomonads combines two c-di-GMP-mediated processes: transcription of a large adhesin and its cell surface display via posttranslational proteolytic control. Here, we characterize an orthologous c-di-GMP effector system and show that it is operational in Vibrio cholerae, where it regulates two distinct classes of adhesins. Through structural analyses, we reveal a conserved autoinhibition mechanism of the c-di-GMP receptor that controls adhesin proteolysis and present a structure of a c-di-GMP-bound receptor module. We further establish functionality of the periplasmic protease controlled by the receptor against the two adhesins. Finally, transcription and functional assays identify physiological roles of both c-di-GMP-regulated adhesins in surface attachment and biofilm formation. Together, our studies highlight the conservation of a highly efficient signaling effector circuit for the control of cell surface adhesin expression and its versatility by revealing strain-specific variations.

## Abstract (precis)

Vibrio cholerae, the causative agent of the diarrheal disease cholera, benefits from a sessile biofilm lifestyle that enhances survival outside the host but also contributes to host colonization and infectivity. The bacterial second messenger c-di-GMP has been identified as a central regulator of biofilm formation, including in V. cholerae; however, our understanding of the pathways that contribute to this process is incomplete. Here, we define a conserved signaling system that controls the stability of large adhesion proteins at the cell surface of V. cholerae, which are important for cell attachment and biofilm formation. Insight into the regulatory circuit underlying biofilm formation may inform targeted strategies to interfere with a process that renders this bacterium remarkably adaptable to changing environments.

## INTRODUCTION

**FIG 1:**
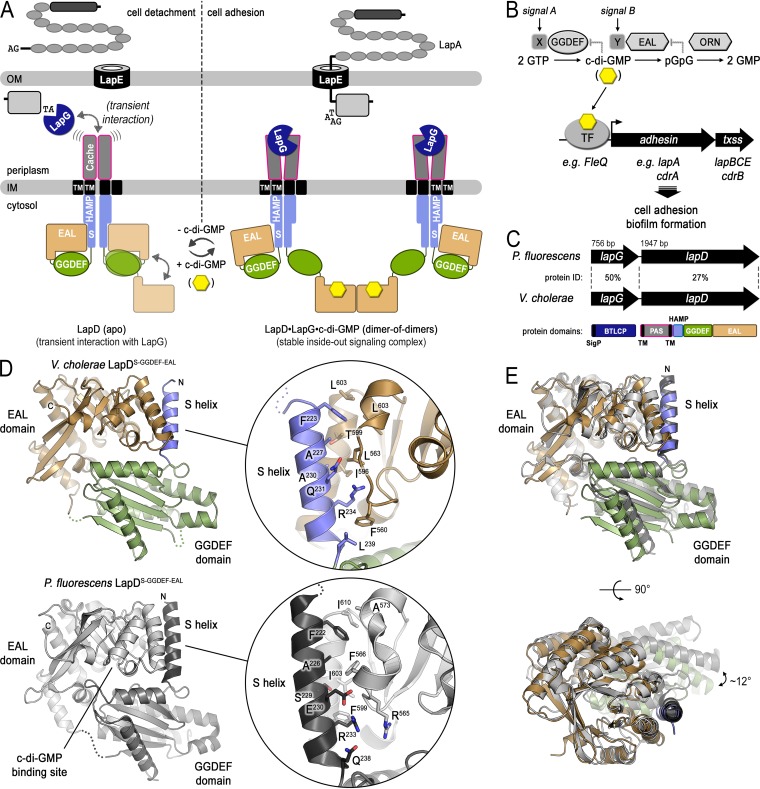
Structure of the autoinhibited cytoplasmic module of V. cholerae LapD. (A) Model of posttranslational LapA regulation via LapDG and c-di-GMP in P. fluorescens. (B) Canonical model for the transcriptional regulation of adhesin proteins via c-di-GMP-responsive transcription factors based on P. fluorescens and P. aeruginosa. (C) Sequence conservation between P. fluorescens LapDG and the putative V. cholerae orthologs. (D) Crystal structure of the S helix-GGDEF-EAL domain module of V. cholerae LapD in the absence of c-di-GMP. The bottom panel shows the corresponding structure of P. fluorescens LapD for comparison. The insets show the residues comprising the S helix-EAL domain interface. (E) Superposition of the cytoplasmic modules of V. cholerae and P. fluorescens LapD in their apo state. Structures were superimposed using the EAL domain as reference. Two orthogonal views are shown.

The biofilm growth mode is a lifestyle preferred by microorganisms (1). Biofilm-forming ability enhances environmental survival, transmission, and infectivity of microbes, including Vibrio cholerae, the causative agent of the severe diarrheal disease cholera (2, 3). Hallmarks of biofilms include interactions of cells with each other, with biotic or abiotic surfaces, and with their surrounding extracellular matrix. For such interactions, bacteria rely on the presence of adhesive, often proteinaceous, appendages on their surface, which include the growing family of repeats-in-toxin (RTX) adhesins that mediate surface attachment of microorganisms (4, 5). Production, cell-surface localization, retention, and regulatory mechanisms for this class of adhesins have been well characterized for a *Pseudomonas* protein, LapA, whose function is controlled by the bacterial second messenger c-di-GMP (Fig. 1A) (6, 7). LapA is transported to the cell surface by a specific type 1 secretion system (T1SS) where it remains anchored in the system’s outer membrane-associated TolC-like subunit LapE (8, 9). In many bacteria, the RTX adhesin gene is linked to a bacterial transglutaminase-like cysteine proteinase (BTLCP), called LapG in Pseudomonas fluorescens (10–13). LapG acts on the transport intermediate, the adhesin spanning the LapE outer membrane channel, by recognizing and cleaving a specific sequence motif between an N-terminal, highly stable retention domain and the membrane-spanning portion of the adhesin (4, 7, 9, 14). LapG-containing operons also harbor a gene that encodes a conserved c-di-GMP receptor, LapD. LapD is composed of a periplasmic Cache domain, flanked by single-transmembrane helices, followed in the cytoplasm by a juxtamembrane HAMP domain with a signaling (or S) helix that leads into a GGDEF and EAL domain module (11, 15–17). GGDEF domains usually function as enzymatic units with diguanylate cyclase activity that produce c-di-GMP from two molecules of GTP (Fig. 1B); EAL domains, on the other hand, act as phosphodiesterases that degrade c-di-GMP to linear di-GMP (18). However, in LapD, both domains are enzymatically inactive, yet the EAL domain retains its ability to bind c-di-GMP (17).

Through detailed structure-function studies, a mechanism for the regulation of LapG via LapD has emerged (Fig. 1A): LapD adopts an autoinhibited conformation that is characterized by the GGDEF domain functioning as a lid atop the EAL domain, which occludes the c-di-GMP binding site (16). This conformation is stabilized by an intramolecular interaction between the S helix of the HAMP domain and the homodimerization interface observed in c-di-GMP-bound EAL domains, thus also preventing EAL domain dimerization (Fig. 1A, left). LapD’s periplasmic domain has a low affinity for the protease LapG in this state, and as a result, LapG can diffuse freely in the periplasm and proteolytically process LapA, releasing the adhesin from the cell surface (16, 19). When c-di-GMP is produced by specific diguanylate cyclases, the second messenger binds to and dimerizes LapD receptors, which increases LapD’s affinity for LapG (Fig. 1A, right) (20–22). LapG that is sequestered to the surface of the inner membrane via LapD is unable to reach and cleave LapA efficiently. Consequently, LapA remains associated with the TolC-like component of the T1SS, which supports cell adhesion (9). The events can be reversed by specific phosphodiesterases that degrade c-di-GMP (23), converting LapD back to an autoinhibited state with low affinity for LapG. Thus, this system controls the reversible adhesion of bacteria through a posttranslational mechanism using c-di-GMP. Closely related, *lapD*-*lapG*-containing operons and corresponding adhesins have been predicted in many proteobacteria (11, 16). Regulatory mechanisms similar in part to those described above have been determined experimentally for orthologous systems in Pseudomonas putida (13, 24), Shewanella oneidensis (25, 26), and Bordetella bronchiseptica (27). In Pseudomonas aeruginosa, the corresponding LapD/LapG pair controls surface anchorage of an unrelated type Vb secretion system comprising the outer membrane transporter CdrB and the adhesin CdrA (11, 28, 29).

In addition to posttranslational regulation, c-di-GMP levels also impact transcriptional control of biofilm matrix formation (6, 30–32) (Fig. 1B). For example, expression of the *cdrAB* operon in P. aeruginosa increases steeply when c-di-GMP is produced by the diguanylate cyclase WspR (28, 33). Likewise, LapA expression in P. putida is controlled by c-di-GMP (34, 35). In both cases, expression is regulated through the conserved AAA^+^ domain-containing transcriptional regulator FleQ that binds c-di-GMP directly, resulting in a structural change and altered DNA binding properties (36, 37).

Genes encoding LapD and LapG orthologs have been predicted to be present in V. cholerae (11, 16) (Fig. 1C). However, the operon containing these putative orthologs lacks genes for adhesins that could be regulated by this system. Based on our previous work on V. cholerae regulatory networks, we surmised that the flagellum-regulated hemagglutinin FrhA and VCA0849 (named c-di-GMP-regulated adhesin A or CraA from here onwards) would be the adhesins for the V. cholerae LapDG/c-di-GMP signaling module (7, 9, 16, 38). FrhA, which belongs to the RTX adhesin family, facilitates attachment of V. cholerae (O1 classical strain) to mammalian cells and chitin beads (38). Expression of *frhA* is regulated by a diguanylate cyclase, CdgD. Likewise, transcriptomic analysis of cells with altered levels of c-di-GMP revealed that expression of *craA*, which encodes a large protein predicted to be secreted by a T1SS, increases at a high level of c-di-GMP (30). Furthermore, *craA* expression is coregulated with biofilm matrix genes and depends on the presence of the biofilm regulator VpsR (39, 40). The role of *craA*’s gene product is unclear; however, its transcriptional regulation suggests a contribution to biofilm formation. Finally, CraA in V. cholerae contains a periplasmic consensus sequence that can be cleaved artificially by P. fluorescens LapG (9, 16), hinting at regulation akin to the LapADG system in pseudomonads, a hypothesis we set out to test in this study.

Here, we characterize the transcriptional and posttranslational regulation of two large adhesins in V. cholerae, FrhA and CraA, by the second messenger c-di-GMP. We present structural insight into the autoinhibition and activation of V. cholerae LapD by c-di-GMP. We also show that the BTLCP/LapG ortholog of this organism functions as a calcium-dependent protease that cleaves FrhA and CraA adhesins at consensus sites. In addition to these conserved features, distinct regulation of the two adhesins by c-di-GMP is apparent at transcriptional and functional levels that are also dependent on the genetic background. Together, our study uncovers an expansive regulatory network for controlling reversible cell adhesion in V. cholerae strains via c-di-GMP.

## RESULTS

### Structural basis for Vibrio cholerae LapD autoinhibition and activation by c-di-GMP.

We previously predicted LapD and LapG orthologs in V. cholerae, albeit with sequence identities of only 27% and 50%, respectively, compared to their P. fluorescens counterpart (Fig. 1C) (11, 16). The low sequence conservation of the LapD ortholog in particular renders predictions regarding the conservation of regulatory features described first for P. fluorescens LapD unreliable. To gain unbiased insight into the structure and regulation of V. cholerae LapD, we determined a crystal structure of its cytoplasmic domains comprising the S helix followed by GGDEF and EAL domains in the absence of c-di-GMP. Crystals diffracted X rays to a maximum resolution of 2.7 Å (see Table S1 in the supplemental material). Phases were obtained by using molecular replacement with the individual GGDEF and EAL domains of the corresponding P. fluorescens structure (PDB code 3pjx [16]) as the search models. The electron density maps resolved the S helix with the final refined model spanning residues 221 to 638 (Fig. 1D, top).

10.1128/mBio.02822-19.1 TABLE S1 Data collection and refinement statistics. Download Table S1, DOCX file, 0.02 MB. Copyright © 2019 Kitts et al. 2019 Kitts et al. This content is distributed under the terms of the Creative Commons Attribution 4.0 International license.

Despite the low sequence identity, the structures of V. cholerae and P. fluorescens LapD superimpose with an overall root mean square deviation (RMSD) of 1.7 Å, indicating structural conservation of their apo states (Fig. 1D and E). The major difference pertains to a rigid body rotation of the GGDEF domain of approximately 12° relative to the EAL domain (Fig. 1E). However, the autoinhibitory S helix-EAL domain interaction responsible for P. fluorescens LapD autoinhibition at low cellular c-di-GMP levels is well preserved in the structure of the V. cholerae ortholog. More specifically, residues F^223^, A^227^, and R^234^ of the S helix (corresponding to F^222^, A^226^, and R^233^ of P. fluorescens LapD) nestle against a partially hydrophobic interface on the EAL domain (Fig. 1D). As seen with P. fluorescens LapD, this contact places the GGDEF domain atop the putative c-di-GMP binding site on the EAL domain.

**FIG 2:**
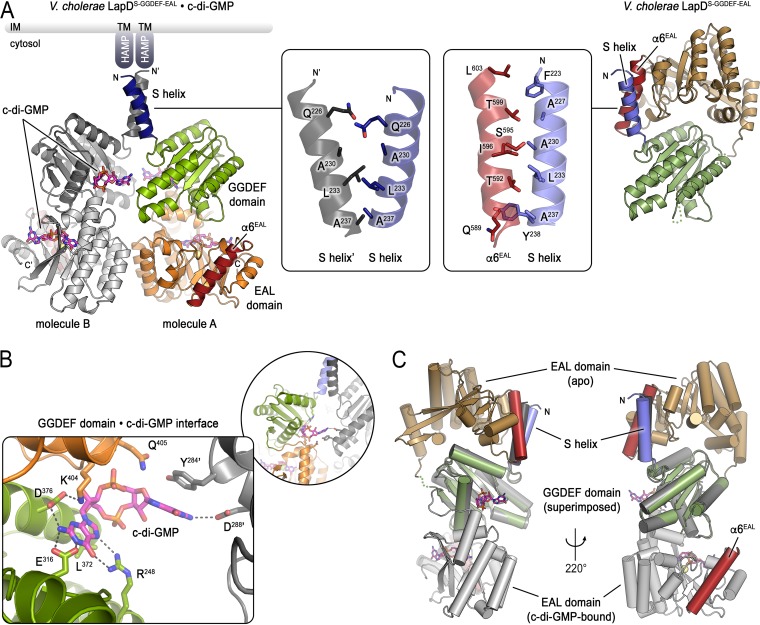
Structure of the c-di-GMP-bound cytoplasmic module of V. cholerae LapD. (A) Comparison between the c-di-GMP-bound and unbound structures of V. cholerae LapD’s cytoplasmic module. The c-di-GMP-bound protein crystallized with two protein molecules per asymmetric unit (left) (molecule A: S helix, dark blue; GGDEF domain, light green; EAL domain, orange with red α6 helix; molecule B: shades of gray). GGDEF and EAL domains bind a single c-di-GMP molecule each. The insets show the S helix dimerization interface in the c-di-GMP-bound dimer (left inset) or the S helix-α6 helix (EAL domain) interface of the apo state (right inset). The apo state is shown for comparison in a similar view as the in the left panel with regard to the S helix-GGDEF domain fragment (right) (S helix, slate; GGDEF domain, dark green; EAL domain, tan with red α6 helix). LapD’s periplasmic domains are not shown. (B) Detailed view of the c-di-GMP binding site on the GGDEF domain of V. cholerae LapD (colored as in panel A, left). (C) Superposition the structures of apo- and c-di-GMP-bound V. cholerae LapD. The respective GGDEF domains were superimposed to highlight the conformational change of this regulatory module. The relative position of the S helix would be fixed by the preceding HAMP and transmembrane domains (not shown). Two views separated by a 220° rotation along the *y* axis are shown. The apo state and c-di-GMP-bound (molecule B) protomers are colored as in panel A.

A high-resolution model for the second messenger-activated state of LapD has been elusive. To provide insight into the conformational changes the cytosolic module undergoes when c-di-GMP binds, we here report a structure of the S helix/GGDEF/EAL domain-containing module of V. cholerae LapD in the presence of c-di-GMP at 2.6 Å resolution (Fig. 2 and Table S1). The asymmetric unit of the crystals contained two molecules of LapD that align with an RMSD of 0.4 Å. A minor difference pertains to the angle at which the S helix projects from the GGDEF domain, which differs by 18° between the two molecules. The two protomers form a dimer via homotypic interactions of their S helices and EAL domains (interface areas: S helices, 343 Å^2^; EAL domains, 525 Å^2^ [41]) (Fig. 2A). The homotypic S helix interaction appears to be rather weak with only two major hydrophobic contacts (L^233^ and A^237^). It remains to be seen whether the helices can pack more tightly, in particular, when the regulatory homodimeric HAMP domain would be present. Based on secondary structure predictions and a recent crystal structure of a HAMP-S helix-containing regulatory module of a bacterial sensor histidine kinase (42), the S helix forms a direct helical extension with the second helix of the HAMP domain, which may establish more favorable helical packing interactions. Hydrophobic residues A^227^, F^223^, and A^230^ of LapD’s S helix are candidate residues for a more extended interaction surface. The GGDEF domains are not involved in intermolecular protein-protein interactions; however, there is a c-di-GMP molecule bound to each of the GGDEF domains that contributes to homodimerization (Fig. 2A and B). This c-di-GMP binding site on V. cholerae LapD’s GGDEF domain is located below the degenerate active site, distinct from nucleotide-binding sites observed in other GGDEF domain structures (see Fig. S1). Several of the residues forming this c-di-GMP binding site are conserved in LapD orthologs (Fig. S1A).

10.1128/mBio.02822-19.4 FIG S1 Sequence conservation and comparison of LapD’s c-di-GMP binding site at the GGDEF domain. (A) Surface sequence conservation of the c-di-GMP binding site in LapD orthologs. A sequence alignment was generated using Clustal Omega (1). Conservation scores were mapped on the solvent-accessible surface of a c-di-GMP-bound LapD monomer. The second molecule in the crystal lattice is shown as a grey ribbon. The inset shows a different view of the binding site without the second LapD molecule shown. (B) Comparison of c-di-GMP binding poses on GGDEF domains. Crystal structures of nucleotide-bound GGDEF domains of LapD, XCC4471 (2), and PleD (3, 4) are shown in the same orientation. Residues involved in c-di-GMP binding to LapD (and their corresponding positions in other GGDEF domains) are shown as sticks. Download FIG S1, DOCX file, 0.8 MB. Copyright © 2019 Kitts et al. 2019 Kitts et al. This content is distributed under the terms of the Creative Commons Attribution 4.0 International license.

A c-di-GMP molecule is also bound to the canonical binding site on the EAL domain. In previously reported c-di-GMP-bound structures, EAL domains homodimerize via a conserved interface involving helix α6 (16, 43–45). In contrast, c-di-GMP binding to V. cholerae LapD appears nonobligatory to such EAL dimerization, since the interface involving helix α6 is involved in neither intradimer contacts nor other packing contacts in the lattice of this crystal form (Fig. 2A). S helix dimerization in the c-di-GMP-bound state utilizes the same face of the helix that rests against the EAL domain in the autoinhibited state (Fig. 2A, insets) (16), suggesting that LapD activation involves a rotation of the cytoplasmic module when c-di-GMP binds to the receptor.

**FIG 3:**
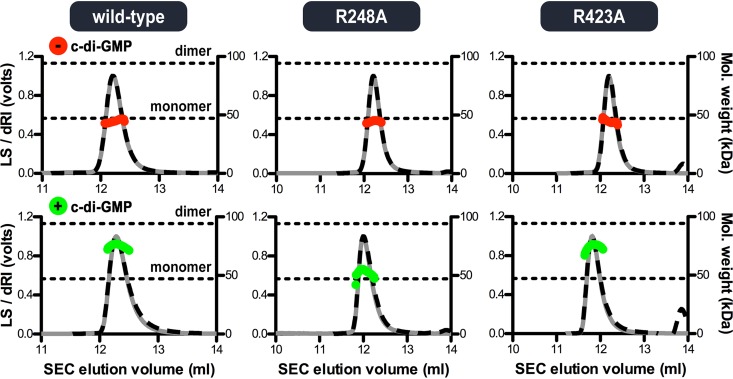
Molecular weight determination shows c-di-GMP-mediated dimerization of the cytoplasmic V. cholerae LapD module. Absolute molecular weights (red and green data points across elution peaks) are plotted on the right axes. Theoretical monomer and dimer molecular weights for the purified S helix-GGDEF-EAL domain-containing LapD fragment are indicated as horizontal dashed lines. Wild-type protein and variants with a point mutation at the c-di-GMP binding site of either the GGDEF (R^248^A) or EAL (R^423^A) domain were analyzed using SEC-MALS (90° light scattering, gray solid lines; refractive index signals, black dashed lines; plotted on the left axis).

We previously used size exclusion chromatography coupled with multiangle light scattering (SEC-MALS) to determine the molar mass and hence oligomerization in solution of the regulatory cytoplasmic module of P. fluorescens LapD, comprised of S helix, GGDEF, and EAL domains (46). The protein is monomeric in the absence of nucleotide but dimerizes when c-di-GMP is included in the mobile phase of the chromatography (16). To assess the impact of c-di-GMP binding to the GGDEF domain and the homotypic S helix interactions on the structure of LapD, we determined the oligomeric states of the corresponding V. cholerae LapD fragment by the same method (Fig. 3). A comparable result was observed with wild-type V. cholerae LapD, where the protein module is monomeric in the absence of c-di-GMP but forms transient dimers when c-di-GMP is included in the buffer. Mutation of a conserved arginine residue that coordinates c-di-GMP at the GGDEF domain to alanine (R^248^A) rendered the protein monomeric even in the presence of c-di-GMP. In contrast, mutation of a major c-di-GMP-interacting residue at the canonical EAL domain binding site (R^423^A) did not affect the oligomerization characteristics of the protein.

Together, our structural analysis revealed that the autoinhibited conformation of LapD is a conserved feature in this class of transmembrane c-di-GMP receptors. Activation by c-di-GMP appears to proceed through a large conformational change during which the EAL domain dislodges from the S helix and moves as a rigid body under the GGDEF domain (Fig. 2C). The orientation of the GGDEF domain relative to the S helix appears invariable, stabilized by a conserved salt bridge (D^240^-R^311^ in V. cholerae LapD) (16). The observed S helix dimerization upon c-di-GMP binding would require a rotation of this module, likely impacting the conformation of the HAMP, transmembrane, and periplasmic domains as a consequence.

### The V. cholerae BTLCP functions as a bona fide LapG ortholog.

**FIG 4:**
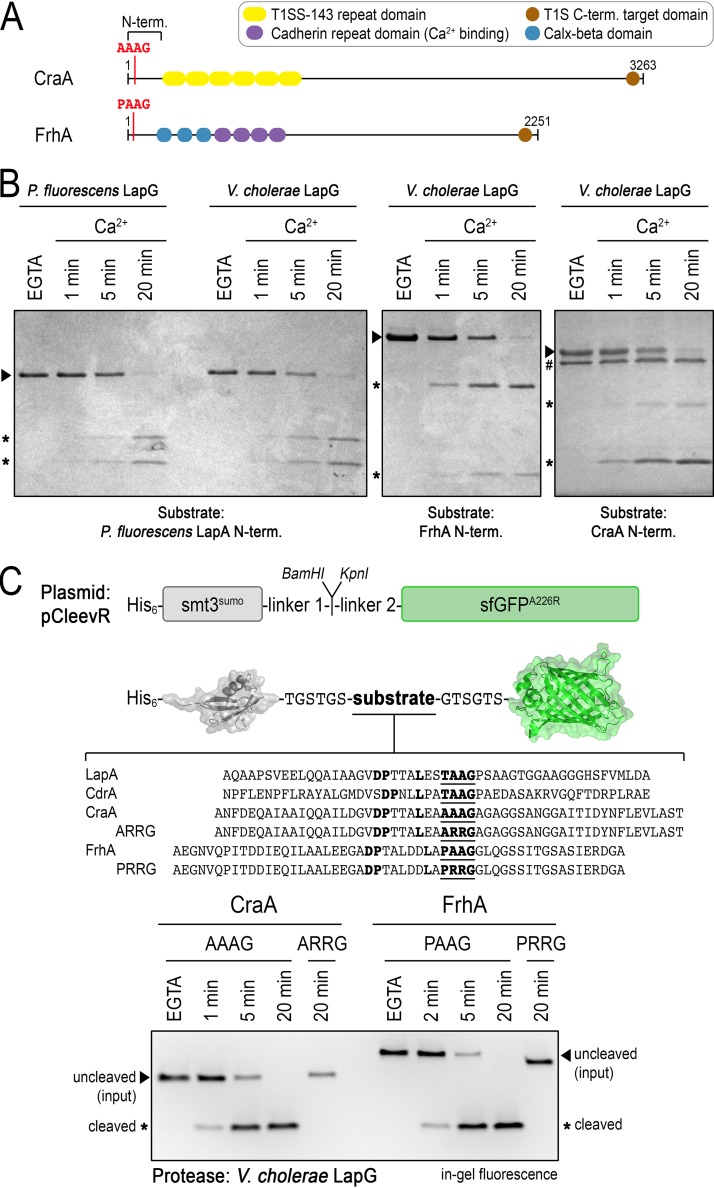
V. cholerae LapG is an active calcium-dependent protease. (A) Domain organization of CraA and FrhA. The N-terminal domain comprises the periplasmic retention module and predicted consensus sequence for cleavage by LapG. (B) V. cholerae LapG proteolyzes the N-terminal fragments of P. fluorescens LapA, FrhA, and CraA in a calcium-dependent manner. Processing of CraA (relative to LapA and FrhA) required 10-fold more LapG (0.5 versus 5 μM LapG; LapG is indicated by # in the gel showing CraA processing). Representative gels from three independent experiments are shown. (C) Characterization of LapG specificity with the pCleevR assay. A diagram is shown of the pCleevR constructs in which various adhesin sequences containing their respective LapG cleavage sites are flanked N terminally by a His6-tagged SUMO protein and C terminally by sfGFP-A^226^R. The SDS-PAGE, imaged by in-gel fluorescence to detect fragments containing the sfGFP module, shows LapG activity toward peptides from CraA and FrhA. Proteolysis was prevented in the presence of EGTA or when the consensus di-alanine motif, the site where LapG cleaves its substrates, was mutated to a di-arginine motif. Representative gels from three independent experiments are shown.

Previous bioinformatic predictions identified two large proteins, VC1620/FrhA and VCA0849/CraA, that contain putative N-terminal periplasmic retention domains followed by sequence motifs resembling LapG substrate sites (9, 11, 16, 47) (Fig. 4A). FrhA and CraA differ in their primary sequences but also the types of repeat domains they display at the cell surface. CraA resembles repeats-in-toxin adhesins (RtxA) and contains at least 6 domains that have been classified as T1SS-143 repeat-containing domains (IPR019959) (48–50). FrhA, on the other hand, encompasses Calx-beta and cadherin repeat domains. Importantly, the purified N-terminal fragment of CraA is cleaved by LapG, a BTLCP from P. fluorescens (9). However, the activity of the BTLCP encoded by the V. cholerae genome has not been validated to date, constituting a gap in our understanding of adhesin processing in this organism.

Here, we compared the activities of BTLCP (or LapG) orthologs from P. fluorescens and V. cholerae on the N-terminal fragments of the various adhesins (Fig. 4B). As a control and as shown previously (11, 19), the P. fluorescens LapA fragment was processed over time to two smaller subfragments upon incubation with substoichiometric amounts of the endogenous LapG in a calcium ion-dependent manner. No proteolysis was observed in the presence of the calcium-chelating agent EGTA. Incubation of LapA with V. cholerae LapG yielded comparable results with regard to cleavage products and calcium dependence. Notably, V. cholerae LapG also processed the N-terminal fragments of FrhA and CraA in a calcium-dependent manner. Under the chosen conditions, FrhA appeared to be a better substrate than CraA, since 10-fold more LapG (500 nM for FrhA compared to 5 μM for CraA) was required to match similar proteolysis kinetics.

To confirm the exact cleavage site in FrhA and CraA, we assayed V. cholerae LapG activity against peptides spanning the predicted consensus protease recognition and cleavage sites, flanked by SUMO and a superfolder green fluorescent protein (sfGFP) variant (11) (Fig. 4C). The SUMO moiety renders the peptide soluble, while sfGFP enables detection of tagged proteins in SDS-PAGE via in-gel fluorescence (51). Peptide selection was guided by our previous work, in which we showed that optimal proteolysis of LapA by LapG is achieved when the consensus sequence is extended N terminally by a cryptic, poorly conserved motif that is predicted to adopt a helical fold (11). Similar to the results obtained with the N-terminal fragments that also included the adhesin’s retention domain, we observed proteolysis of the FrhA and CraA peptides over time when calcium was available but no cleavage in the presence of EGTA. In contrast to the previous experiment using the N-terminal fragments, V. cholerae LapG cleaved FrhA and CraA peptides with similar efficiencies. LapG is predicted to cleave a di-alanine sequence, and mutation of the motif PAAG to PRRG or AAAG to ARRG in FrhA or CraA, respectively, renders the peptides insensitive to LapG.

In summary, we show that the BTLCP gene adjacent to V. cholerae *lapD* encodes an active, calcium-dependent LapG-like protease with specificity for both FrhA and CraA, despite variations in the consensus substrate sequences (Fig. 4C).

### Expression of *frhA* and *craA* is regulated by changes in c-di-GMP levels.

Our previous studies suggest that expression of *frhA* is modulated by CdgD, a diguanylate cyclase (38), and that *craA* expression is under the control of an established c-di-GMP-dependent regulatory system (30, 39). Here, we built on the earlier reports and extend our investigation into the transcriptional regulation of the two distinct V. cholerae adhesins, particularly with respect to c-di-GMP signaling.

**FIG 5:**
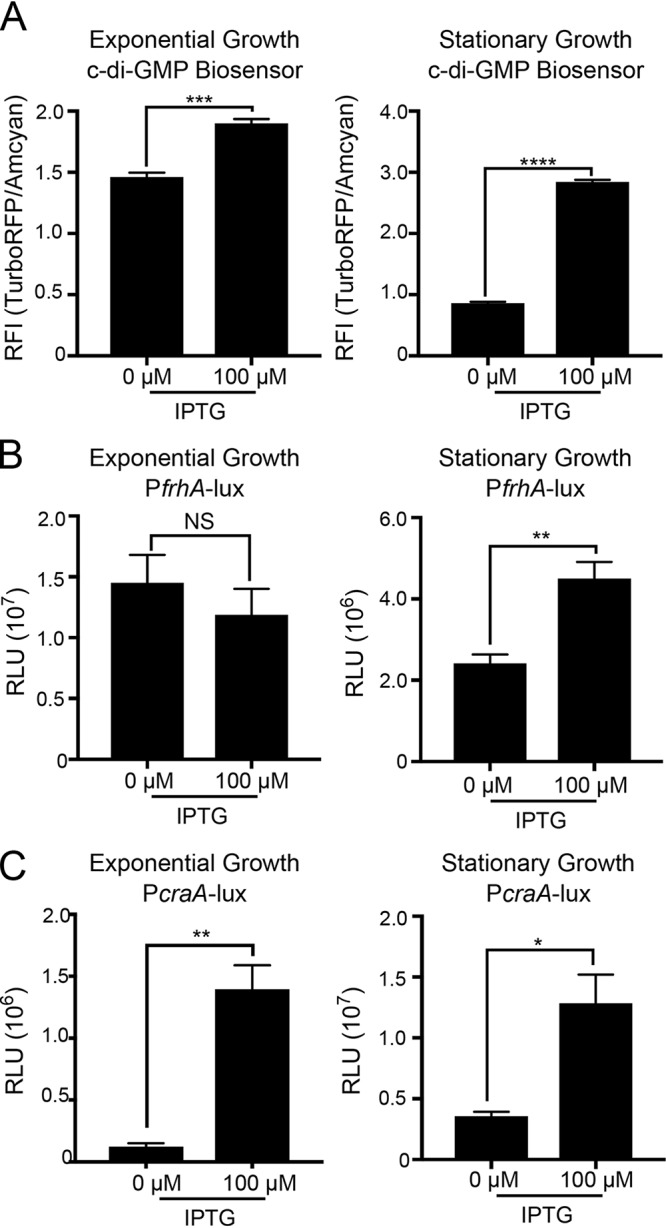
Changes in c-di-GMP levels affect expression of *frhA* and *craA* in the strain Ptac-*cdgF*. (A) Production of c-di-GMP in strain Ptac-*cdgF* was measured during exponential (left) and stationary (right) phases of growth in the absence or presence of 100 μM IPTG using a c-di-GMP reporter. Fluorescence intensity (FI) of AmCyan is used as a normalizer and FI of TurboRFP is an indicator of c-di-GMP level. Expressions of the transcriptional fusions P*frhA*-lux (B) and P*craA*-lux (C) were analyzed during exponential (left) and stationary (right) phases of growth in a genetic background where c-di-GMP levels are modulated using inducer IPTG (strain Ptac-*cdgF*). Cells were grown in the absence or presence of 100 μM IPTG. The graphs represent the means and standard deviations of the relative fluorescent intensity (RFI) (FI TurboRFP/AmCyan) or relative luminescence units (RLU) from at least three independent biological replicates. Means were compared using an unpaired *t* test with Welch’s correction. Mean differences with a *P* value of ≤0.05 were deemed significant. *, *P* ≤ 0.05; **, *P* ≤ 0.01; ***, *P* ≤ 0.001; ****, *P* ≤ 0.0001; NS, not significant.

To provide better insights into c-di-GMP-mediated regulation of FrhA and CraA, we first measured expression of *frhA* and *craA* using transcriptional reporters in a V. cholerae strain in which c-di-GMP production is controlled through expression of a diguanylate cyclase CdgF (VCA0956) from an isopropyl β-d-1-thiogalactopyranoside (IPTG)-inducible promoter (PlacIq-*lacI*-Ptac-*cdgF*) (52). We determined the relative levels of c-di-GMP in strains harboring Ptac-*cdgF* grown to exponential or stationary phase by using a fluorescence-based ratiometric c-di-GMP reporter (53). The relative fluorescence intensity (RFI) values obtained with this reporter are directly proportional to c-di-GMP levels (53, 54). Cells grown to exponential or stationary phase in the presence of 100 μM IPTG showed a 1.3- and 3.3-fold increase in c-di-GMP levels, respectively, compared to those in uninduced cells (Fig. 5A). These results showed that c-di-GMP levels are significantly higher in the presence of induction and that the difference in c-di-GMP levels between uninduced and induced cells is more dramatic in stationary-phase-grown cells.

We next analyzed *frhA* and *craA* expression using P*frhA*-*lux* and P*craA*-*lux* in cells grown to exponential or stationary phase in the absence or presence of inducer. While *frhA* expression was unaffected in exponentially grown cells, *frhA* expression showed a 1.9-fold increase in cells grown to stationary phase in the presence of inducer in comparison to that in uninduced cells (Fig. 5B). In contrast, c-di-GMP-dependent expression of *craA* was higher in cells grown either to exponential or stationary phase (an increase of 3.6- or 11.3-fold, respectively, compared to that in uninduced cells) (Fig. 5C). These results indicate that c-di-GMP levels positively regulate expression of *frhA* in stationary phase and of *craA* in both exponential and stationary phases.

### c-di-GMP receptors regulate transcription of *frhA* and *craA*.

**FIG 6:**
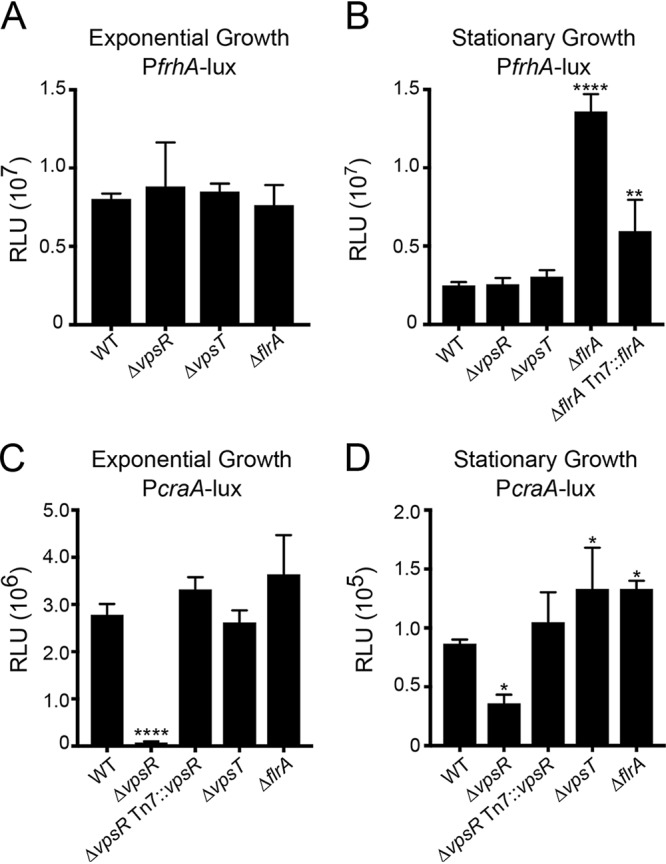
Different c-di-GMP receptors modulate expression of *frhA* and *craA*. Expressions of the transcriptional fusion P*frhA*-*lux* (A and B) and P*craA*-*lux* (C and D) were analyzed during exponential (left) and stationary (right) phases of growth in the indicated strains. The graphs represent the means and standard deviations of the relative luminescence units (RLU) from at least three independent biological replicates. Means were compared to WT using a one-way ANOVA and Dunnett’s multiple-comparison test. Mean differences with an adjusted *P* value of ≤0.05 were deemed significant. *, *P* ≤ 0.05; **, *P* ≤ 0.01; ****, *P* ≤ 0.0001.

c-di-GMP regulates transcription via specific c-di-GMP receptors. In V. cholerae, the transcriptional regulators VpsR, VpsT, and FlrA have been shown to bind to c-di-GMP (32, 55, 56) and regulate gene expression. Therefore, we analyzed the expression of the transcriptional fusions P*frhA*-*lux* and P*craA*-*lux* in wild-type (WT) and Δ*vpsR*, Δ*vpsT*, and Δ*flrA* strains (Fig. 6). Expression of *frhA* was comparable in all strains when the cells were grown to exponential phase (Fig. 6A). In cells grown to stationary phase, *frhA* expression was unaltered in Δ*vpsR* and Δ*vpsT* strains. In contrast, we observed a 5.5-fold increase of expression in the Δ*flrA* strain (Fig. 6B). Introduction of a wild-type copy of *flrA* at the Tn*7* site of the Δ*flrA* strain partially complemented this phenotype, showing only a 2.4-fold increase in *frhA* expression compared to that in the wild-type strain.

In exponentially grown cells, expression of *craA* decreased 39-fold in the Δ*vpsR* strain compared to that in the wild-type strain (Fig. 6C). This decrease in expression of *craA* was fully complemented when *vpsR* was expressed in *trans*. The expression of *craA* in Δ*vpsT* and Δ*flrA* strains was unaffected under these conditions. In cells grown to stationary phase, *craA* expression decreased 2.3-fold in the Δ*vpsR* strain compared to that in the wild-type strain, and this defect was complemented by the expression of *vpsR* in *trans* (Fig. 6D). We also found expression of *craA* was increased (1.5-fold) in both the Δ*vpsT* and Δ*flrA* strains. (Fig. 6D). Taken together, these results show that VpsR is the main activator of *craA* expression. The roles of VpsT and FlrA are less clear and appear to depend on the growth phase.

We did not observe an effect of the absence of either VpsR or VpsT on *frhA* expression at basal levels of c-di-GMP. We speculated that the activity of these regulators on *frhA* expression might require elevated c-di-GMP levels. Hence, we analyzed expression of these genes in a rugose (R) strain of V. cholerae that is documented to have higher cellular c-di-GMP levels (57) and in the corresponding RΔ*vpsR*, RΔ*vpsT*, RΔ*vpsRT*, and RΔ*flrA* strains (see Fig. S2). In cells grown to exponential phase, *frhA* expression did not change in RΔ*vpsR*, RΔ*vpsRT*, and RΔ*flrA* strains compared to that in the rugose strain (Fig. S2A). However, we observed a 2-fold increase in *frhA* expression in the RΔ*vpsT* strain (Fig. S2A). In cells grown to stationary phase, we did not observe any difference in *frhA* expression in RΔ*vpsR*, RΔ*vpsT*, and RΔ*vpsRT* strains (Fig. S2B). In the RΔ*flrA* strain during stationary phase, *frhA* expression was increased 1.8-fold. These results corroborate FlrA as a repressor of *frhA* expression during stationary-phase growth.

10.1128/mBio.02822-19.5 FIG S2 VpsR and VpsT play different roles in the regulation of *frhA* and *craA* in the rugose phase variant of strain A1552. Expressions of the transcriptional fusion P*frhA*-*lux* (A and B) and P*craA*-*lux* (C and D) in rugose genetic backgrounds during exponential (left) and stationary (right) phases of growth. The graphs represent the means and standard deviations of the relative luminescence units (RLU) from at least three independent biological replicates. Means were compared to rugose strain using a one-way ANOVA and Dunnett’s multiple-comparison test. Mean differences with an adjusted *P* value of ≤0.05 were deemed significant. **, *P* ≤ 0.01; ****, *P* ≤ 0.0001. Download FIG S2, DOCX file, 0.06 MB. Copyright © 2019 Kitts et al. 2019 Kitts et al. This content is distributed under the terms of the Creative Commons Attribution 4.0 International license.

In cells grown to exponential phase, expression of *craA* decreased 163-fold in the RΔ*vpsR* strain, 2-fold in the RΔ*vpsT* strain, and 149-fold in the RΔ*vpsR* Δ*vpsT* strain compared to that in the rugose strain (Fig. S2C). Similar changes to *craA* expression were observed in cells grown to late stationary phase, where expression of *craA* decreased 48-fold in the RΔ*vpsR* strain, 3-fold in the RΔ*vpsT* strain, and 48-fold in RΔ*vpsR* Δ*vpsT* strain (Fig. S2D). These results corroborate the role of VpsR as the main regulator of *craA* and show that VpsT acts as a positive regulator of *craA* in the rugose strains with high c-di-GMP levels.

### The LapDG/c-di-GMP signaling module regulates hemagglutination.

**FIG 7:**
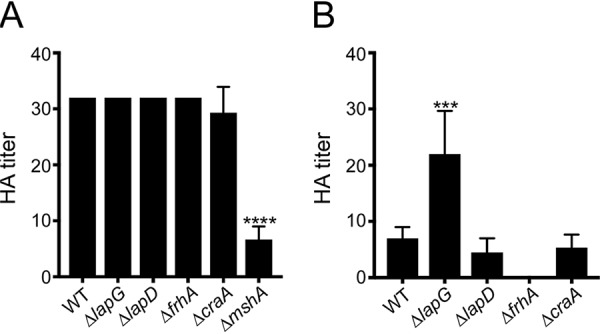
The LapDG system regulates hemagglutination. Hemagglutination assays were performed with V. cholerae O1 El Tor A1552 (A) or O1 classical O395 (B) strains. Either wild-type (WT) or indicated deletion mutants were used. The reciprocal of the lowest fold dilution (hemagglutinin [HA] titer) at which cells were able to agglutinate red blood cells was recorded. The graphs represent the means and standard deviations of HA titers from at least three biological replicates. One-way ANOVA and Dunnett’s multiple-comparison test were used to compare the mean of each mutant to WT. Mean differences with a *P* value of ≤0.05 were deemed significant. ***, *P* ≤ 0.001; ****, *P* ≤ 0.0001.

Since FrhA supports hemagglutination (38) and, as we show above, is a target of LapG *in vitro* (Fig. 4), we investigated next whether the V. cholerae LapDG system impacts hemagglutination of human red blood cells (Fig. 7). We observed no deficiencies in hemagglutination with the Δ*lapG*, Δ*lapD*, and Δ*craA* mutants but also not with a Δ*frhA* mutant (Fig. 7A). One reason for this apparent discrepancy to the previous report (38) regarding the involvement of *frhA* in this process pertains to the strain background. There are more than 200 serogroups of V. cholerae, and they differ in the expression of virulence factors, surface antigens, and other factors. The V. cholerae O1 serogroup is further divided into classical and El Tor biotypes. Involvement of FrhA in hemagglutination was established in V. cholerae O1 classical strain O395, while our experiments were carried out in V. cholerae O1 El Tor strain A1552. Notably, in V. cholerae El Tor strains, mannose-sensitive hemagglutinin (MSHA), a type 4 pilus, is involved in hemagglutination (58, 59). MSHA pili are assembled on the surfaces of V. cholerae cells of the O1 El Tor strain but not the O1 classical strain. A Δ*mshA* mutant in the El Tor strain A1552 background was included as a control, which showed significantly reduced hemagglutination (Fig. 7A), confirming that MSHA pili are the dominant adhesion structure driving the hemagglutination phenotype in this strain. We next examined the hemagglutination abilities of the Δ*lapG*, Δ*lapD*, Δ*craA*, and Δ*frhA* mutants generated in the V. cholerae O1 classical strain O395 (Fig. 7B). The absence of the LapG protease led to increased hemagglutination by 3.1-fold, while the absence of the LapD receptor led to a 1.6-fold decrease. In this genetic background, red blood cell hemagglutination was abolished in the Δ*frhA* strain. Without LapD, LapG will cleave outer membrane-anchored adhesins constitutively, while a LapG deletion prevents release of the adhesins from the cell surface, explaining the inverse phenotypes of the two mutants observed in the hemagglutination assay. Taken together, these results show that the V. cholerae LapDG/c-di-GMP signaling module controls the presentation of FrhA on the cell surface and, in turn, red blood cell hemagglutination. However, this phenotype is dependent upon the genetic background, likely impacted by the expression of other adhesin-type proteins such as MSHA pili.

### The LapDG/c-di-GMP signaling module regulates biofilm formation in V. cholerae.

**FIG 8:**
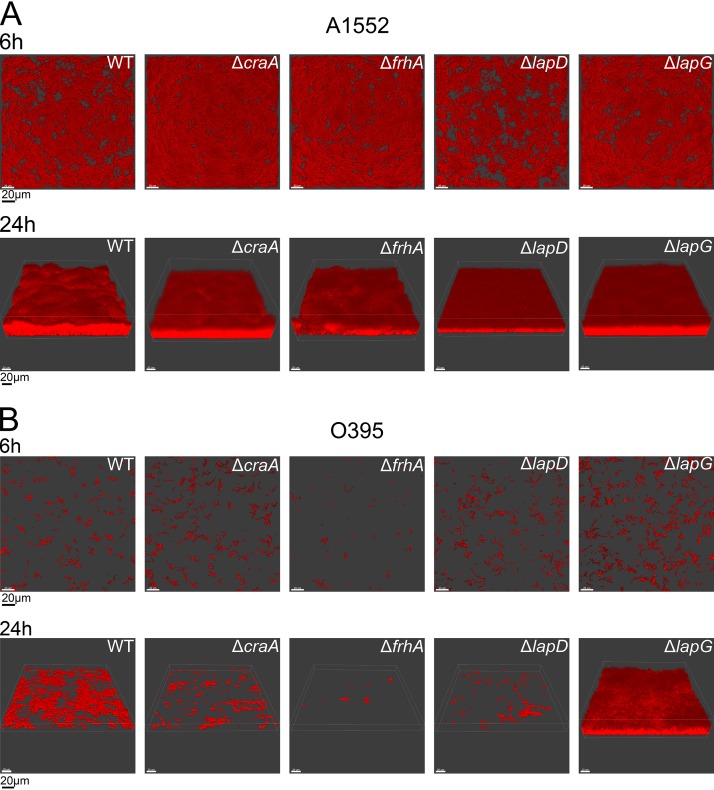
The LapDG system regulates biofilm formation. Biofilm formation was analyzed using once-through flow cell system using wild-type (WT) and indicated mutants in V. cholerae O1 El Tor A1552 (A) or V. cholerae O1 classical O395 (B) strains. COMSTAT analysis of the biofilms is provided in Table S2 in the supplemental material.

c-di-GMP is a key signaling molecule controlling biofilm formation, and LapDG signaling modules are critical for biofilm formation in P. fluorescens, P. putida, and P. aeruginosa (11, 13, 17, 19, 29). Thus, we next asked whether this function is conserved in V. cholerae. In particular, we analyzed the contributions of LapD, LapG, FrhA, and CraA to V. cholerae biofilm formation when cells were grown under flow conditions. In the V. cholerae O1 classical strain O395, we observed markedly different biofilm formation dynamics for the strains lacking the LapDG signaling module and associated adhesins (Fig. 8A). Deletion of LapG and LapD had inverse effects, showing increased and decreased biofilm formation, respectively. FrhA appeared to contribute more prominently to biofilm formation of this strain than CraA. Quantitative analysis of biofilm formation was performed using COMSTAT to determine biomass, average/maximum thickness, and substrate coverage (see Table S2). For the Δ*lapG* strain at 24 h, we found that biomass, average thickness, and substratum coverage were 8.3-, 2.1-, and 2.5-fold greater, respectively, than in the wild-type strain. In contrast, in the Δ*lapD* strain, we observed 14.9-, 1.2-, and 14.9-fold lower biomass, thickness, and substratum coverage, respectively, than in the wild type. While both adhesins contribute to biofilm formation, trends that FrhA deletion has a more pronounced effect on biofilm formation than a CraA deletion are also reflected in this quantitative analysis (fold decreases in biomass, thickness, and coverage in the Δ*frhA* strain: 42.9, 1.3, and 41.9, respectively; fold decreases in biomass, thickness, and coverage in the Δ*craA* strain: 6.4, 1.2, and 5.0, respectively).

10.1128/mBio.02822-19.2 TABLE S2 COMSTAT analysis of biofilm properties. Download Table S2, DOCX file, 0.02 MB. Copyright © 2019 Kitts et al. 2019 Kitts et al. This content is distributed under the terms of the Creative Commons Attribution 4.0 International license.

In the V. cholerae O1 El Tor strain A1552, biofilm formation defects were less pronounced than those in the V. cholerae O1 classical strain O395. In the A1552 strain, only the lack of LapG resulted in a significant change in biofilm properties, where the Δ*lapG* mutant formed biofilms with increased surface colonization and small but reproducible increases in biomass and thickness (Fig. 8B and Table S2). These results indicate that V. cholerae biofilm formation is modulated by the LapDG signaling module and its associated adhesions but that strain differences contribute to the relative importance of these adhesion systems for biofilm formation, mirroring the results from the hemagglutination assay.

**FIG 9:**
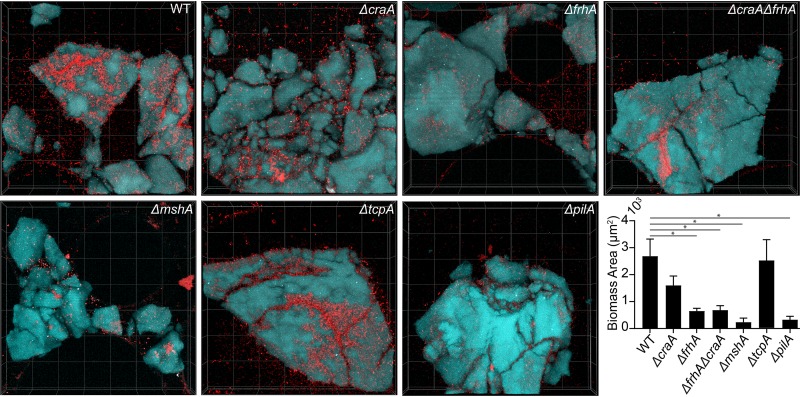
Δ*craA* and Δ*frhA* mutants in V. cholerae O1 El Tor A1552 show attachment defects on chitin. All images are top down 3D renderings with dimensions of 212 μm by 212 μm by 33 μm (length by width by depth); red indicates cellular biomass and cyan shows the chitin particles. Quantification of the biomass in of each strain was performed after 48 h of growth of 3 biological replicates. Wilcoxon signed-ranks tests were performed to compare each mutant to the wild type (WT). *, *P* < 0.05. All other mutants shown were found to not be significantly different from WT.

Though biofilm formation on glass is a very fruitful environment for studying biomass accumulation and architectural development, V. cholerae forms biofilms on numerous other surfaces in natural contexts (3, 60). As the differences in biofilm formation for Δ*craA* and Δ*frhA* backgrounds relative to that for the wild type were only modest on glass for V. cholerae O1 El Tor A1552, we wondered if the contributions of the FrhA and CraA adhesins to biofilm formation would be more evident on chitin, a surface actively sought by V. cholerae in aquatic environments. To occupy and successfully digest chitin for consumption via exoenzyme secretion, V. cholerae must produce biofilms on particles of chitin polymer (61). To test for biofilm growth on chitin, we used a customized microfluidic assay in which pieces of chitin (sterilized shrimp shell) were immobilized, colonized with V. cholerae inoculum, and perfused with a defined seawater medium (61, 62). Attachment and subsequent biofilm growth on the chitin can then be quantified using confocal microscopy (Fig. 9). As shown previously (63, 64), *ΔmshA* and *ΔpilA* strains had dramatic 10-fold reductions in chitin attachment in the A1552 El Tor background. We found that on chitinous surfaces, the Δ*frhA* and Δ*craA* strains showed 4.2-fold and 2-fold reduced biomass, respectively, compared to that of the A1552 wild-type strain. A double deletion strain of both Δ*frhA* and Δ*craA* showed biomass accumulation (3.9-fold reduction) and a visual phenotype indistinguishable from those of the Δ*frhA* single deletion mutant. These results suggest that both adhesins play a role in initial biofilm formation of V. cholerae on chitin in its natural marine environment.

## DISCUSSION

Biofilm formation is a multifactorial and highly regulated process. A central modulator of biofilm formation in a diverse group of microorganisms is c-di-GMP. In this study, we characterized a conserved c-di-GMP receptor, LapD, and its associated periplasmic protease, LapG, and established the pair’s role in controlling the stability of two c-di-GMP-regulated V. cholerae adhesins, FrhA and CraA. We also demonstrated that the V. cholerae LapG ortholog is an active calcium-dependent protease that processes both FrhA and CraA (Fig. 4), thus controlling cell adhesion and biofilm formation.

Based on structural work, we show that the Vibrio cholerae LapD ortholog adopts a similar autoinhibited conformation utilizing the same intramolecular interfaces as determined for the related P. fluorescens LapD, despite low sequence conservation (Fig. 1). We also determined for V. cholerae LapD the hitherto elusive structure of this cytoplasmic module bound to c-di-GMP, which identified a second c-di-GMP binding site on the GGDEF domain in addition to the canonical site located on the EAL domain (Fig. 2). While the noncanonical site appears to be crucial for V. cholerae LapD dimerization of the GGDEF-EAL domains (Fig. 3), its exact function remains to be determined. With regard to the canonical site, c-di-GMP binding does not appear to establish a conformation that supports EAL domain dimerization via the α6 helix-containing interface that has been observed in other LapD and EAL domain protein structures (16, 43–45). Instead, the backsides of the EAL domains contact each other, exposing the c-di-GMP binding site of the EAL domain to the cytosol. Whether this feature correlates with c-di-GMP binding to the GGDEF domain requires further investigation.

The c-di-GMP-bound structure of V. cholerae LapD is consistent with solution scattering-based modeling of full-length P. fluorescens LapD, which suggested a conformation with an exposed EAL domain that, in the fully activated state, can bridge two LapD dimers through EAL domain dimerization (see model in Fig. 1A) (21). But even in the context of P. fluorescens LapD, EAL domain dimerization via the canonical interface is puzzling: it clearly occurs in solution with the isolated EAL domain and the full-length receptor, but biofilm phenotypes of mutant receptors indicate an ancillary role in the regulation of LapD *in vivo* (16). It is quite possible the phenotypes are complex, since mutations that destabilize the EAL domain dimer are likely to also impact the main autoinhibitory interface between the S helix and the EAL domain (16, 21). A cytosol-facing EAL domain may also enable interactions with other proteins, for example, diguanylate cyclases that produce c-di-GMP (21, 65, 66). Such protein-protein interactions between enzymes and c-di-GMP-binding receptors have been shown to facilitate signaling specificity in complex signaling networks (67, 68).

A second level of c-di-GMP regulation in this system pertains to transcription of the adhesin proteins. We show that transcription of *frhA* and *cra*A is governed by c-di-GMP-dependent transcriptional activators FlrA, VpsT, and VpsR. In this context, it is important to note that cellular c-di-GMP in V. cholerae is modulated by the growth state; exponentially grown cells have higher c-di-GMP levels than those in stationary phase (69). Transcription of *craA* is strongly induced by c-di-GMP regardless of the growth stage. Expression of *craA* depends on the presence of VpsR but is mostly independent of VpsT and FlrA.

In the V. cholerae classical strain O395, expression of *frhA* has been shown to be regulated as a class 4 gene from the flagellar regulatory hierarchy (38). The expression of *frhA* was downregulated in the absence of the flagellar regulators (FlrA, FlrBC, and FliA) as well as in the absence of another flagellum-regulated gene, *cdgD*, which encodes a diguanylate cyclase that negatively impacts motility (38, 54). It is, however, unclear how this c-di-GMP-synthesizing enzyme regulates *frhA* expression. In the El Tor strain A1552, we found that expression of *frhA* is upregulated by the absence of the master regulator of flagellar genes, FlrA, only during late stationary phase. It has yet to be determined if FlrA can directly regulate *frhA* or if it acts indirectly. Coincidently, expression of *frhA* was upregulated by increased c-di-GMP levels only during late stationary phase. Since FlrA is allosterically inhibited by c-di-GMP, it is possible that high c-di-GMP levels partially release *frhA* transcription from the negative control exerted by FlrA during late stationary phase.

The V. cholerae A1552 rugose genetic background is a phase variant locked in a mode of constitutively high c-di-GMP and biofilm matrix production. In the rugose genetic background, VpsR and VpsT are strongly activated by c-di-GMP and, in turn, activate the transcription of multiple regulatory targets (40). One of these targets is *craA*, which has a highly conserved VpsR binding site 222 bp upstream of its translational start site (TTTCACAATTGAGA) (70). *frhA*, on the other hand, appears to be negatively regulated by VpsT but not by VpsR in this genetic background. The extent by which VpsR and VpsT act independently is yet to be fully determined. It has been shown that when c-di-GMP levels are elevated, VpsT can antagonize the negative regulation of a few targets of H-NS (70, 71). It is possible that in the rugose background, VpsT releases the repression of a negative regulator of *frhA* that may be under the control of H-NS or a similar nucleoid-associated protein. The reported consensus sequence for VpsT binding is not present in the regulatory region of *frhA*. This observation does not rule out a direct involvement of VpsT, since its binding sites tend to be variable (70, 72). Together, these observations suggest that the cell has evolved mechanisms to limit *frhA* expression under conditions where *craA* and biofilm matrix gene expression are elevated.

Another layer of control arises from a recent chromatin immunoprecipitation sequencing (ChIP-seq) experiment that revealed cAMP receptor protein (CRP) binding to the regulatory region of *frhA*, *craA*, and *vpsT*—genes that were previously found to be regulated by CRP in a whole-genome expression analysis study designed to identify the CRP regulon (73, 74). It is possible that some of the variations in expression of these genes, and especially *frhA*, involve changes in cAMP and CRP abundance and/or CRP activity. In addition, *frhA* expression is also repressed by the transcriptional regulator TfoY (75). TfoY expression itself is controlled by at least two mechanisms, involving either a Vc2 c-di-GMP binding riboswitch or transcriptional control via the c-di-GMP binding protein VpsR, depending on the cellular c-di-GMP levels (76).

Regarding posttranslational regulation of the adhesins, we found that the N-terminal domains of CraA and FrhA were cleaved by V. cholerae LapG, which strongly suggests that release of these proteins from the cell surface functions in analogy to the system encoded by the *lap* operon in P. fluorescens (6, 7). The mechanisms by which FrhA and CraA are secreted and retained on the cell surface of V. cholerae are not well understood. CraA is related to P. fluorescens LapA. Secretion of LapA is linked to a specific T1SS that anchors the adhesin at the cell surface. In P. fluorescens, the genes encoding LapA, LapG, LapD, and the specialized T1SS (LapE, LapB, and LapC) are located in close proximity. The gene encoding FrhA (VC1620) in V. cholerae is in close proximity only to the gene VC1621, which encodes a LapE-like protein that in P. fluorescens forms the conduit for anchoring LapA at the cell surface (7, 9). Orthologues of *lapG*, *lapD*, *lapB*, and *lapC* are located in the second chromosome of V. cholerae (VCA1081, VCA1082-3, VCA1084, and VCA1080, respectively). The genomic context of *craA* (VCA0849) includes a diguanylate cyclase (VCA0848), which could be functionally linked to this c-di-GMP-regulated adhesin, and genes predicted to encode ABC-type transport proteins (VCA0854 and VCA0855) but lacks a gene encoding a LapE-like protein. Whether FrhA and CraA utilize the same T1SS for transport and outer membrane anchorage or adhesin-specific systems requires further characterization.

We found that the LapDG system as well as FrhA and CraA plays a role in biofilm formation in a strain- and surface-specific manner (Fig. 8 and 9; see also Table S2 in the supplemental material). Functionally, the lack of FrhA prevents the V. cholerae O1 classical strain from forming biofilms or hemagglutinating red blood cells. The lack of CraA in the O1 classical strain has a negative impact on biofilm formation but does not affect hemagglutination. The absence of the protease LapG results in a marked increase in biofilm formation and hemagglutination compared to that for the wild type, indicating the regulation of the two adhesins via this protease *in vivo*. In contrast, in the El Tor strains, the contributions of the LapDG system and FrhA and CraA adhesins to biofilm formation were more evident when biofilms were grown on chitin, the natural substratum and growth substrate for *Vibrio* spp. in aquatic environments.

Differences in the regulation of the same gene in the classical versus El Tor strains are not uncommon, and perhaps, in this particular case, these differences have been selected due to particular requirements for the *frhA* product for the survival and/or adaptation of these two strains. It is important to bear in mind that MSHA pili are not produced in the classical strain O395. As a result, FrhA and perhaps other adhesins are needed to substitute for MSHA function in this strain. Furthermore, *Vibrio* strains can differ with regard to the numbers of repeat units in their adhesins (e.g., in the case of FrhA), which also could contribute to differences in surface adhesion. The nature of the surface to which V. cholerae cells adhere may dictate what combination of adhesins need to be used in order to attach strongly enough to be able to colonize the surface. For instance, it is not known if c-di-GMP, FrhA, and/or CraA contribute to the enhanced attachment and growth on chitinous surfaces of the filamentous V. cholerae O139 strain CVD112 (62). Another open question pertains to the relevance of the distinct domain architecture of the various adhesins, which could add another layer of regulation, for example, through calcium binding to FrhA’s extracellular domains. In analogy to the adhesin P. aeruginosa CdrA interacting with Psl polysaccharide, which enhances stability and packing of the biofilm matrix (77), one may also speculate that V. cholerae adhesins engage in cooperative interactions with each other or *Vibrio* polysaccharide (Vps). In the future, it will be interesting to elucidate the relative or distinct contributions of Vibrio cholerae adhesins and their molecular regulation in the context of different strains of this biomedically relevant organism. Strain differences concerning adhesion factors may shape microbial fitness, function, and/or ecology, thus contributing to specific host-microbe interactions.

## MATERIALS AND METHODS

### Bacterial strains, plasmids, and culture conditions.

The strains and plasmids used in this study are listed in Table S3 in the supplemental material. Escherichia coli CC118λpir strains were used for DNA manipulation, and E. coli S17-1 (λ *pir*) and SM10 (λ *pir*) strains were used for conjugation with V. cholerae. V. cholerae and E. coli strains were grown aerobically at 30°C and 37°C, respectively, unless otherwise stated. Cultures were grown in lysogeny broth (LB) (10 g/liter tryptone, 5 g/liter yeast extract, 10 g/liter NaCl [pH 7.5]). LB agar medium contains granulated agar (BD Difco, Franklin Lakes, NJ) at 1.5% (wt/vol). Antibiotics and inducers were used, when necessary, at the following concentrations: ampicillin (Ap), 100 μg/ml; kanamycin (Kan), 50 μg/ml; rifampin (Rif), 100 μg/ml; gentamicin (Gm), 15 μg/ml; chloramphenicol (Cm), 20 μg/ml for E. coli and 5 μg/ml for V. cholerae; and isopropyl β-d-1-thiogalactopyranoside (IPTG), 0.1 mM. For large-scale protein expression, E. coli BL21(DE3) strains (New England BioLabs, Ipswich, MA) were grown in Terrific broth (TB) (24 g/liter yeast extract, 20 g/liter tryptone, 17 mM KH_2_PO_4_, 72 mM K_2_HPO_4_, 8 ml/liter glycerol) (BD Difco, Franklin Lakes, NJ).

10.1128/mBio.02822-19.3 TABLE S3 Strains and plasmids. Download Table S3, DOCX file, 0.03 MB. Copyright © 2019 Kitts et al. 2019 Kitts et al. This content is distributed under the terms of the Creative Commons Attribution 4.0 International license.

### Recombinant DNA techniques and plasmid and strain constructions.

DNA manipulations were carried out by standard molecular techniques according to the manufacturers’ instructions. Restriction and DNA modification enzymes were purchased from New England BioLabs (Ipswich, MA). PCRs were carried out using primers purchased from ELIM Biopharmaceuticals, Inc. (Hayward, CA) and the PfuUltra II Fusion HS DNA polymerase (Agilent Technologies, Santa Clara, CA). Sequences of the primers used in the present study are available upon request. Plasmid sequences were verified via DNA Sanger sequencing (Cornell Genomics Facility, Ithaca, NY). Site-directed point mutations were introduced using a QuikChange II kit (Agilent Technologies, Santa Clara, CA).

Plasmids were constructed using standard molecular cloning techniques or the Gibson assembly recombinant DNA technique (New England BioLabs, Ipswich, MA). In-frame gene deletions were generated through allelic exchange of a native open reading frame (ORF) with the truncated ORF, as described previously (78). Both fluorescent protein-tagged and chromosomal complementation strains were generated through a Tn*7*-based system that inserts in the genomic region between loci VC0487 and VC0488, as described previously (78). Triparental conjugation was performed, with donor E. coli S17-1 (λ *pir*) cells carrying either pGP704::Tn*7*-GFP (or desired promoter-gene in place of GFP), as well as a second helper E. coli S17-1 (λ *pir*) harboring pUX-BF13 carrying the Tn*7* transposase gene. Transconjugants were selected for on thiosulfate-citrate-bile salts-sucrose (Difco) agar medium containing 15 μg/ml gentamicin at 30°C. Tn*7* insertion strains were verified by PCR analysis. Transcriptional fusions were generated by cloning the upstream regulatory region (∼300 to 500 bp) of genes of interest into the promoterless pBBR*lux* plasmid.

For c-di-GMP induction assays, allelic exchange was used to introduce an IPTG-inducible promoter in place of the native promoter of the diguanylate cyclase VCA0956. The promoter swap was conducted in strain A1552 (this genetic variant is designated Ptac-*cdgF*). A plasmid-based dual-fluorescent c-di-GMP biosensor was used for c-di-GMP measurements. A Pbe promoter regulated constitutive expression of an AmCyan-Bc3-5-TurboRFP cassette, in which Bc3-5 is a triple-tandem c-di-GMP-sensing riboswitch adapted from that described in reference 53 that enables TurboRFP expression in the presence of c-di-GMP. AmCyan is used as a normalizer. A toxin-antitoxin system (hok-sok) was introduced to the plasmid to remove the need for antibiotics during the assays (54).

### Protein expression and purification.

DNA fragments encoding the soluble portion of the V. cholerae LapD ortholog (residues 221 to 636 or 23 to 636 of VCA1082/1083) were amplified from V. cholerae chromosomal DNA by PCR and cloned into a pET28-based vector, which adds an N-terminal Ulp1-cleavable His_6_ SUMO tag. E. coli BL21(DE3) cells harboring the expression plasmid were grown shaking in in Terrific broth (TB) medium (12 g/liter tryptone, 24 g/liter yeast extract, 5 g/liter glycerol, 0.017 M KH_2_PO_4_, and 0.072 M K_2_HPO_4_) supplemented with 50 μg/ml kanamycin at 37°C. When cultures reached an optical density at 600 nm (OD_600_) of 0.8, the temperature was reduced to 18°C and protein expression was induced by addition of 0.5 mM IPTG. Proteins were expressed for 16 h, after which, cells were harvested by centrifugation, resuspended in nickel-nitrilotriacetic acid (Ni-NTA) buffer A (25 mM Tris-HCl [pH 8.5], 500 mM NaCl, and 20 mM imidazole), and flash frozen in liquid nitrogen. A DNA fragment encoding the V. cholerae BTLCP protein lacking the signal peptide (residues 54 to 220 of VCA1081) was amplified from V. cholerae genomic DNA, cloned into the pET28a derivative, and expressed as described above.

DNA fragments encoding segments of VCA0849 (CraA) and VC1620 (FrhA) containing the putative LapG cleavage sites (Fig. 4C) were amplified from V. cholerae genomic DNA and cloned into the pCleevR plasmid (11). Expression from the pCleevR construct creates fusion protein with an N-terminal His_6_ SUMO domain and a C-terminal superfolder green fluorescent protein variant (sfGFP-A^226^R) tag used for in-gel visualization of cleavage reactions (51). Fragments corresponding to the predicted N-terminal retention domain and the LapG cleavage sites of CraA and FrhA (residues 1 to 212 of CraA and 1 to 210 of FrhA) were amplified from genomic DNA and cloned into pET21 or the pET28 derivative, respectively. A corresponding expression plasmid for P. fluorescens LapA (LapA N terminus) was described previously (11, 19). Adhesin proteins fragments were expressed as described above.

For purification of proteins expressed in E. coli, frozen cell suspensions were thawed, and cells were lysed by sonication. After centrifugation, clarified lysates were incubated with Ni-NTA Superflow resin (Qiagen, Hilden, Germany) that was equilibrated with Ni-NTA buffer A. The resin was washed with 20 column volumes of buffer A, followed by elution of protein using 3 column volumes of Ni-NTA buffer B (Ni-NTA buffer A supplemented with 300 mM imidazole [pH 7.5]). Eluted proteins were buffer exchanged into Ulp-buffer (25 mM Tris-HCl [pH 7.5], 500 mM NaCl, 20 mM imidazole) using a fast-desalting column (GE Healthcare Life Science, Marlborough, MA). For BTLCPs and LapD orthologs, Ulp1 (a sumo protease) was added, followed by incubation at 4°C overnight. The reaction was subjected to a second Ni-NTA column step with the flowthrough containing the Ulp1-cleaved untagged target proteins. All proteins were concentrated using Amicon Ultra filters (Millipore, Burlington, MA) with a 10-kDa cutoff, and concentrated protein was subjected to size exclusion chromatography on a Superdex 200 column (GE Healthcare Life Science, Marlborough, MA) that was equilibrated in gel filtration buffer (25 mM Tris-HCl [pH 7.5], 150 mM NaCl). Purified proteins were concentrated using 10-kDa-cutoff Amicon Ultra filters, flash frozen in liquid nitrogen, and stored at −80°C.

### Crystallization, data collection, and structure solution.

Protein crystals of LapD^220–636^ were obtained by hanging-drop vapor diffusion, mixing equal volumes (1 μl) of protein (concentrations of 200 to 650 μM) and reservoir solution. For apo-LapD^220–636^, the reservoir solution consisted of 2.5 M sodium acetate trihydrate, pH 7.0. For c-di-GMP-bound LapD^220–636^, the reservoir solution consisted of 0.2 M sodium formate, 0.1 M bicine (pH 8.5), 20% (wt/vol) polyethylene glycol (PEG) MME 5000, and 0.5 to 1 mM c-di-GMP (Biolog, Bremen, Germany). Crystals were cryoprotected by soaking them in their respective reservoir solutions supplemented with 15% glycerol (apo) or 20% xylitol (c-di-GMP bound), followed by flash freezing and storage in liquid nitrogen. Data were collected at beamline A1 at the Cornell High Energy Synchrotron Source (CHESS; Cornell University, Ithaca, NY).

Data reduction was carried out with the software package HKL2000 (79) or iMosflm/Aimless (80, 81). Phases for the initial structure were obtained from molecular replacement using the software package Phenix (82) with the isolated GGDEF and EAL domains from the crystal structure of P. aeruginosa LapD (PDB ID 3pjw [16]) as the search models. Refinement in Phenix and COOT (83) yielded the final models. Data collection and refinement statistics are summarized in Table S1. Structural illustrations were made in PyMOL (Schrödinger, LLC, New York, NY). All crystallographic software was distributed by SBGrid (84).

### Size exclusion chromatography coupled with multiangle light scattering.

Molecular masses of LapD protein samples were determined using size exclusion chromatography coupled with static multiangle light scattering (SEC-MALS) (46). Purified protein at concentrations between 1 and 15 mg/ml (20 to 300 μM) was subjected to gel filtration on a Bio Sep-SEC-s 3000 column (Phenomenex, Torrance, CA) that was equilibrated in MALS buffer (25 mM Tris-HCl [pH 7.5], 150 mM NaCl). For samples with c-di-GMP in the mobile phase, 50 μM c-di-GMP was added to the MALS buffer. The SEC was coupled to a static 18-angle light-scattering detector (DAWN HELEOS-II) and a refractive index detector (Optilab T-rEX; Wyatt Technology, Goleta, CA). Data were collected at 25°C each second for 30 min at a flow rate of 1 ml/min. Data analysis was carried out using the program ASTRA V. The detectors were normalized using a sample of 5 mg/ml bovine serum albumin (BSA) (monomeric fraction; Sigma-Aldrich, St. Louis, MO).

### LapG proteolysis of substrates.

To assess LapG cleavage of purified peptide substrates (expressed from the pCleevR plasmid as His_6_-SUMO-peptide-sfGFP fusion proteins), 5 μM BTLCP/LapG was preincubated for 10 min with either 20 mM EGTA or CaCl_2_ in reaction buffer containing 25 mM Tris HCl (pH 7.5) and 150 mM NaCl (11, 51). Reactions were initiated by addition of BTLCP (0.5 μM final concentration) into 20 μM pCleevR substrates in reaction buffer. Reactions were quenched at 2, 5, and 20 min by mixing an aliquot directly into SDS sample buffer (without sample boiling), and products were analyzed by SDS-PAGE. Gels were imaged by fluorescence (detecting folded sfGFP) using a ChemiDoc imager (Bio-Rad, Hercules, CA). Similar conditions were used for the protease assays with the N-terminal adhesin fragments as the substrates, except for VC0849, for which 5 μM (final concentration) LapG was used. N-terminal adhesin fragments and their proteolytic products were resolved by SDS-PAGE followed by Coomassie staining.

### c-di-GMP biosensor assay.

The V. cholerae strain Ptac-*cdgF* harboring a c-di-GMP dual-fluorescent biosensor was grown aerobically overnight in LB supplemented with gentamicin. Overnight cultures were diluted 1:200 into fresh LB with or without 0.1 mM IPTG. Cells were grown aerobically at 30°C to exponential phase (OD_600_ of 0.3 to 0.4), and then fluorescence was measured using a PerkinElmer Victor3 multilabel counter (PerkinElmer, Waltham, MA). Another fluorescence measurement was taken in stationary phase. Fluorescence is reported as relative fluorescence intensity (RFI) (FI TurboRFP/FI Amcyan). Assays were repeated for a minimum of three independent biological replicates, with three technical replicates measured for all assays. Statistical significance was determined using an unpaired *t* test with Welch’s correction.

### Luminescence assay.

V. cholerae strains harboring transcriptional reporters were grown aerobically overnight in LB supplemented with 5 μg/ml chloramphenicol. Overnight cultures were diluted 1:200 into fresh LB containing chloramphenicol. When the strain Ptac-*cdgF* was used, cells were grown in the presence or absence of 0.1 mM IPTG. Cells were grown aerobically at 30°C to exponential phase (OD_600_ of 0.3 to 0.4), and luminescence was measured using a PerkinElmer Victor3 multilabel counter (PerkinElmer, Waltham, MA). Another luminescence measurement was taken in stationary phase. Luminescence expression is reported as relative luminescence units (RLU; counts min^−1 ^ml^−1^/OD_600_ unit). Assays were repeated for a minimum of three independent biological replicates, with three technical replicates measured for all assays. For Ptac-*cdgF* experiments, statistical significance was determined using an unpaired *t* test with Welch’s correction.

### Hemagglutination assay.

Hemagglutination of V. cholerae strains was tested using human red blood cells (RBCs) (R407-0050; Rockland Immunochemicals, Limerick, PA). Ten percent human RBCs were centrifuged (2,000 × *g*, 5 min, 4°C) and washed with gentle resuspension in Krebs-Ringer Tris (KRT) buffer (7.5 g/liter NaCl, 0.383 g/liter KCl, 0.318 g/liter MgSO_4_·7H_2_O, and 0.305 g/liter CaCl_2_ in 10 mM Tris-HCl [pH 7.4]) until the supernatant was clear. RBCs were gently resuspended in KRT buffer to a final concentration of 2% (vol/vol). RBCs were kept on ice throughout. Overnight cultures of V. cholerae strains were diluted 1:200 in fresh LB and grown with aeration to an OD_600_ of 0.3 to 0.4. Five milliliters of each culture was centrifuged (4,000 × *g*, 10 min, 4°C), washed twice in KRT buffer with resuspension, and concentrated 10-fold in 500 μl KRT buffer. Round-bottom 96-well plates were used for the hemagglutination assay. Two technical replicates were performed per biological replicate. The top row of each plate was left blank of bacterial cells and was used as a negative control. Two hundred microliters of KRT buffer was added to A1, while 200 μl of the 10-fold concentrated V. cholerae cells were added to wells B1 to H1, with a different strain for each row. One hundred microliters of KRT buffer was added to all other wells in the plate. Two-fold serial dilutions were performed horizontally across the plate. For column 12, 100 μl was discarded, resulting in an equal volume per well across the entire 96-well plate. Seventy-five microliters of the 2% human RBCs was added rapidly to each well of the plate with minimal pipetting to prevent RBC lysis. The plates were incubated at 4°C overnight, and images were taken the next morning. The last dilution not showing clear RBC button formation at the bottom of the well was used as the hemagglutination titer for that strain. One-way analysis of variance (ANOVA) and Dunnett’s multiple-comparison test were used for determination of statistical significance.

### Flow cell biofilm experiments and confocal laser scanning microscopy.

Overnight cultures of *gfp*-tagged V. cholerae strains were diluted 1:200 and used as flow cell inoculum. One hundred fifty microliters of diluted cells was injected into an Ibidi μ-Slide VI 0.4 (Ibidi 80606; Ibidi LLC, Verona, WI) and left for 1 h at room temperature with no flow to allow adherence to the substratum. A flow of 10% LB (with full strength NaCl) was initiated at a rate of 4.5 ml/h. Confocal laser scanning microscopy (CLSM) images of the biofilms were captured with an LSM 880 (Zeiss, Jena, Germany), using an excitation wavelength of 488 nm and an emission wavelength of 543 nm. Three-dimensional (3D) images of the biofilms were processed using Imaris software (Bitplane, Zurich, Switzerland). All image processing parameters in Imaris (opacity, min, max, etc.) were kept identical for each set of images for accurate comparison. Quantitative analysis was done using COMSTAT2.1 (http://www.comstat.dk) (85). For calculation of substrate coverage with z-stack images, the highest substratum coverage from the bottom 20 stacks was chosen, representing the surface of the slide.

### Chitin microfluidic assay.

Microfluidic devices bonded to size 1.5 36-mm by 60-mm coverslips were created using standard soft lithography techniques (86). A columnar chamber design was used to trap the chitin flakes within the device. To place chitin in the device as well as establish flow, the same techniques were used as described in reference 62. Briefly, chitin was placed in the chamber under a high flow rate, and then a syringe pump was used to perfuse the device with defined seawater medium. Strains were grown overnight at 37°C in shaking culture and then normalized to an OD_600_ of 1 prior to inoculation. Chambers were then inoculated with culture and allowed to rest for 30 min. After this time, a flow regime of 0.2 μl/min was established, and biofilms were allowed to grow for 48 h prior to imaging. Biomass was quantified using custom MATLAB scripts (MathWorks, Natick, MA) as in references 61, 62, and 87.

### Data availability.

The atomic coordinates and structure factors have been deposited in the Protein Data Bank (https://www.rcsb.org/) under PDB identifier (ID) codes 6PWJ and 6PWK.
