# Supplementary material for: A Conserved Regulatory Circuit Controls Large Adhesins in Vibrio cholerae
Source: mBio. 2019 Dec 3;10(6):e02822-19. doi: 10.1128/mBio.02822-19 (PMC6890996; doi:10.1128/mBio.02822-19)
Supplement: TABLE S1 [file mBio.02822-19-st001.docx]

| **Table S1. Data collection and refinement statistics.^a^** | | |
| --- | --- | --- |
| ***Structure*** | LapD*_Vc_* S-GGDEF-EAL (apo) | LapD*_Vc_* S-GGDEF-EAL (c-di-GMP-bound) |
| ***Data collection*** | | |
| Wavelength | 0.6309 | 0.977 |
| Resolution range | 12.7 - 2.70 (2.80 - 2.70) | 33.8 - 2.61 (2.70 - 2.61) |
| Space group | P 32 2 1 | P 21 |
| Unit cell a/b/c (Å) | 129.8 129.8 114.4 | 77.4 90.2 82.8 |
| Unit cell a/b/g (°) | 90 90 120 | 90 115.5 90 |
| Total reflections | 255,233 (26,112) | 117,298 (11,788) |
| Unique reflections | 30,101 (2,856) | 31,030 (3,102) |
| Multiplicity | 8.5 (8.8) | 3.8 (3.8) |
| Completeness (%) | 93.1 (95.1) | 99.9 (100.0) |
| I/sigma(I) | 18.58 (4.18) | 20.89 (1.97) |
| Wilson B-factor | 45.17 | 69.27 |
| CC1/2 | 0.997 (0.877) | n.d. |
| ***Refinement*** | | |
| Reflections (R-free) | 1,974 (197) | 1,999 (188) |
| R-work | 0.2000 (0.2548) | 0.1930 (0.3013) |
| R-free | 0.2434 (0.3234) | 0.2458 (0.3766) |
| Non-hydrogen atoms | 6509 | 6651 |
| macromolecules | 6486 | 6425 |
| Ligands/solvent | 12/11 | 186/40 |
| Protein residues | 815 | 811 |
| Rms (bonds) | 0.004 | 0.004 |
| Rms (angles) | 0.88 | 0.99 |
| Ramachandran |  |  |
| favored (%) | 98.13 | 97.38 |
| allowed (%) | 1.87 | 2.37 |
| outliers (%) | 0.00 | 0.25 |
| Rotamer outliers (%) | 1.45 | 4.68 |
| Average B-factor | 53.55 | 80.26 |
| macromolecules | 53.60 | 80.83 |
| Ligands/solvent | 40.94/37.76 | 63.11/68.77 |
| ^a^Statistics for the highest-resolution shell are shown in parentheses. | | |
