# Supplementary material for: A Conserved Regulatory Circuit Controls Large Adhesins in Vibrio cholerae
Source: mBio. 2019 Dec 3;10(6):e02822-19. doi: 10.1128/mBio.02822-19 (PMC6890996; doi:10.1128/mBio.02822-19)
Supplement: FIG S1 [file mBio.02822-19-sf001.docx]

**
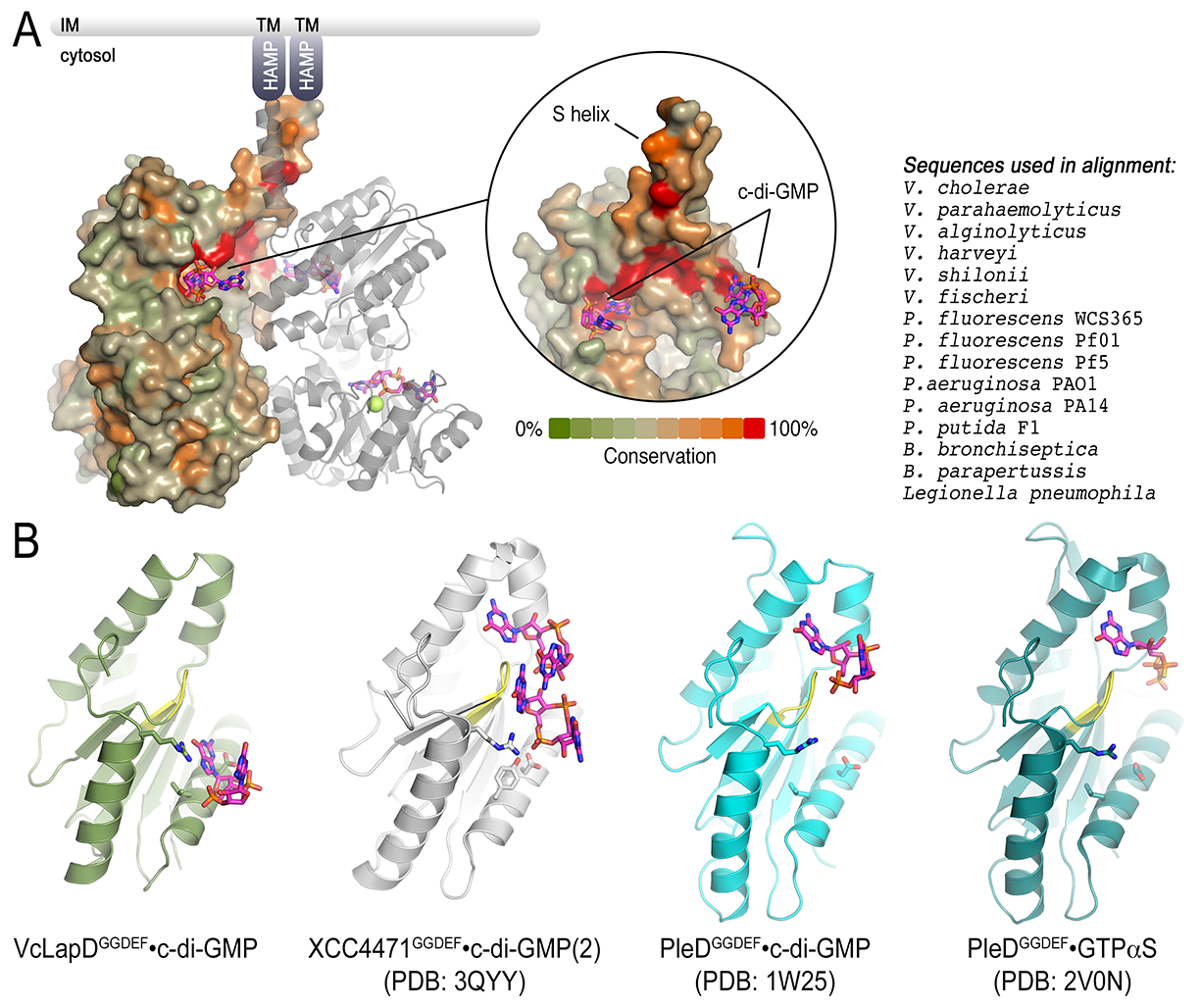
**

1. Madeira F, Park YM, Lee J, Buso N, Gur T, Madhusoodanan N, Basutkar P, Tivey ARN, Potter SC, Finn RD, Lopez R. 2019. The EMBL-EBI search and sequence analysis tools APIs in 2019. Nucleic Acids Res 47:W636-W641.

2. Yang CY, Chin KH, Chuah ML, Liang ZX, Wang AH, Chou SH. 2011. The structure and inhibition of a GGDEF diguanylate cyclase complexed with (c-di-GMP)(2) at the active site. Acta Crystallogr D Biol Crystallogr 67:997-1008.

3. Chan C, Paul R, Samoray D, Amiot NC, Giese B, Jenal U, Schirmer T. 2004. Structural basis of activity and allosteric control of diguanylate cyclase. Proc Natl Acad Sci U S A 101:17084-9.

4. Wassmann P, Chan C, Paul R, Beck A, Heerklotz H, Jenal U, Schirmer T. 2007. Structure of BeF^3-^ -modified response regulator PleD: implications for diguanylate cyclase activation, catalysis, and feedback inhibition. Structure 15:915-27.
