# Supplementary material for: A Conserved Regulatory Circuit Controls Large Adhesins in Vibrio cholerae
Source: mBio. 2019 Dec 3;10(6):e02822-19. doi: 10.1128/mBio.02822-19 (PMC6890996; doi:10.1128/mBio.02822-19)
Supplement: TABLE S2 [file mBio.02822-19-st002.docx]

| **Table S2. COMSTAT analysis of biofilm properties.^a^** | | | | |
| --- | --- | --- | --- | --- |
|  | **Biomass** | **Mean Thickness (SD)** | **Mean Thickness (SD)** | **Substrate coverage** |
|  | (µm^3/µm^2) | Average (µm) | Maximum (µm) | (%) |
| ***Strain A1552 (6h post-inoculation)*** | | | | |
| Wild-type (WT) | 9.502 (1.574) | 11.84 (2.869) | 18.13 (1.847) | 96.66 (3.107) |
| Δ*craA* | 10.92 (1.687) | 12.32 (2.259) | 17.66 (2.178) | 99.25 (0.6337) |
| Δ*frhA* | 10.61 (1.344) | 12.19 (2.143) | 17.66 (2.52) | 98.87 (1.471) |
| Δ*lapD* | 7.673 (1.107) | 9.698 (2.038) | 15.49 (1.724) | 90.28 (5.433) |
| Δ*lapG* | 9.281 (0.9804) | 10.67 (1.624) | 16.63 (1.366) | 97.24 (0.9996) |
| ***Strain A1552 (24h post-inoculation)*** | | | | |
| Wild-type (WT) | 33.64 (4.356) | 37.18 (4.621) | 48.11 (5.199) | 99.29 (1.229) |
| Δ*craA* | 35.45 (4.762) | 40.91 (7.663) | 54.79 (8.325) | 99.55 (1.338) |
| Δ*frhA* | 30.49 (4.363) | 36.15 (6.715) | 47.28 (8.081) | 98.32 (3.113) |
| Δ*lapD* | 32.65 (4.133) | 38.12 (6.425) | 44.87 (6.047) | 99.99 (0.007818) |
| Δ*lapG* | 38.19 (2.692) | 42.24 (1.43) | 52.53 (2.157) | 99.96 (0.1019) |
| ***Strain O395 (6h post-inoculation)*** | | | | |
| Wild-type (WT) | 0.02868 (0.009032) | N/A | N/A | 2.868 (0.9032) |
| Δ*craA* | 0.05002 (0.01081) | N/A | N/A | 5.002 (1.081) |
| Δ*frhA* | 0.007258 (0.002674) | N/A | N/A | 0.7259 (0.2676) |
| Δ*lapD* | 0.0283 (0.01866) | N/A | N/A | 2.83 (1.866) |
| Δ*lapG* | 0.1346 (0.03898) | N/A | N/A | 13.46 (3.898) |
| ***Strain O395 (24h post-inoculation)*** | | | | |
| Wild-type (WT) | 2.805 (1.108) | 9.689 (1.499) | 18.12 (3.013) | 37.22 (12.28) |
| Δ*craA* | 0.439 (0.1966) | 8.376 (1.498) | 16.99 (2.861) | 7.505 (2.182) |
| Δ*frhA* | 0.06544 (0.303) | 7.482 (3) | 13.57 (4.328) | 0.8886 (0.3321) |
| Δ*lapD* | 0.1888 (0.1163) | 8.22 (1.096) | 15.92 (2.357) | 2.506 (1.624) |
| Δ*lapG* | 23.35 (9.486) | 20.19 (8.751) | 31.57 (7.311) | 94.08 (5.065) |
| ^a^Mean values and (SD) are listed. | | | | |
