# Supplementary material for: A Conserved Regulatory Circuit Controls Large Adhesins in Vibrio cholerae
Source: mBio. 2019 Dec 3;10(6):e02822-19. doi: 10.1128/mBio.02822-19 (PMC6890996; doi:10.1128/mBio.02822-19)
Supplement: TABLE S3 [file mBio.02822-19-st003.docx]

| **Table S3. Strains and plasmids.** | | |
| --- | --- | --- |
| **Strain or plasmid** | **Relevant properties** | **Source** |
| ***E. coli* strains** |  |  |
| CC118λ*pir* | Δ(*ara-leu*)*araD* Δ*lacX74 galE galK phoA20 thi-1 rpsE rpoB argE*(Am) *recA1* λ*pir* | (1) |
| SM10λ*pir* | thi thr leu tonA lacY supE recA (RP4-2-Tc::Mu) λpirR6K Km^r^ π^+^ | (2) |
| S17-1λ*pir* | Tp^r^ Sm^r^ *recA thi pro* r_K_^−^ m_K_^+^ RP4::2-Tc::MuKm Tn*7* λ*pir* | (2) |
| ***V. cholerae* strains** |  |  |
| FY_Vc_1 | *Vibrio cholerae* O1 El Tor A1552, wild type, Rif^r^ | (3) |
| FY_Vc_2 | *Vibrio cholerae* O1 El Tor A1552, Rugose variant, Rif^r^ | (3) |
| FY_Vc_514 | *Vibrio cholerae* O395 Classical Biotype, Δ*lacZ,* Sm^r^ | (4) |
| FY_Vc_2272 | FY_Vc_1 ΔvpsR (VC0665), Rif^r^ | (5) |
| FY_Vc_99 | FY_Vc_1 ΔvpsT (VCA0952), Rif^r^ | This study |
| FY_Vc_6284 | FY_Vc_1 Δ*flrA* (VC2137), Rif^r^ | This study |
| FY_Vc_4850 | FY_Vc_1 Δ*frhA* (VC1620), Rif^r^ | This study |
| FY_Vc_2262 | FY_Vc_1 Δ*craA* (VCA0849), Rif^r^ | This study |
| FY_Vc_8222 | FY_Vc_1 Δ*lapG* (VCA1081), Rif^r^ | This study |
| FY_Vc_8225 | FY_Vc_1 Δ*lapD* (VCA1082-83), Rif^r^ | This study |
| FY_Vc_12502 | FY_Vc_1 Δ*mshA* (VC0409), Rif^r^ | This study |
| FY_Vc_12504 | FY_Vc_1 Δ*pilA* (VC2423) | This study |
| FY_Vc_12506 | FY_Vc_1 Δ*tcpA* (VC0828) | This study |
| FY_Vc_14119 | FY_Vc_1 ΔvpsR Tn7::*vpsR*, Rif^r^ | This study |
| FY_Vc_14177 | FY_Vc_1 Δ*flrA* Tn7::*flrA*, Rif^r^ | This study |
| FY_Vc_9917 | Ptac-*cdgF*, Insertion of the P_lacIq_-*lacI* and P_tac_ elements in front of VCA0956 in FY_Vc_1, Rif^r^ | (6) |
| FY_Vc_237 | FY_Vc_1 Tn*7::gfp* Rif^r^ Gm^r^ | (7) |
| FY_Vc_14140 | FY_Vc_1 Δ*frhA* (VC1620 Tn*7::gfp,* Rif^r^ Gm^r^ | This study |
| FY_Vc_1008 | FY_Vc_1 Δ*craA* (VCA0849) Tn*7::gfp,* Rif^r^ Gm^r^ | This study |
| FY_Vc_14143 | FY_Vc_1 Δ*lapG* (VCA1081) Tn*7::gfp,* Rif^r^ Gm^r^ | This study |
| FY_Vc_14146 | FY_Vc_1 Δ*lapD* (VCA1082-83) Tn*7::gfp,* Rif^r^ Gm^r^ | This study |
| FY_Vc_5 | FY_Vc_2 ΔvpsT (VCA0952) Δ*lacZ* Rif^r^ | (8) |
| FY_Vc_6 | FY_Vc_2 ΔvpsR (VC0665) Δ*lacZ* Rif^r^ | (8) |
| FY_Vc_7 | FY_Vc_2 ΔvpsR (VC0665) ΔvpsT (VCA0952) Δ*lacZ* Rif^r^ | (8) |
| FY_Vc_15537 | FY_Vc_2 ΔflrA (VC2137) | This study |
| FY_Vc_12114 | FY_Vc_514 Δ*frhA* (VC1620), Sm^r^ | This study |
| FY_Vc_12119 | FY_Vc_514 Δ*craA* (VCA0849), Sm^r^ | This study |
| FY_Vc_11863 | FY_Vc_514 Δ*lapG* (VCA1081), Sm^r^ | This study |
| FY_Vc_12120 | FY_Vc_514 Δ*lapD* (VCA1082-83), Sm^r^ | This study |
| FY_Vc_13508 | FY_Vc_514 Tn*7::gfp* Sm^r^ Gm^r^ | This study |
| FY_Vc_12181 | FY_Vc_514 Δ*frhA* (VC1620) Tn*7::gfp,* Sm^r^ Gm^r^ | This study |
| FY_Vc_12183 | FY_Vc_514 Δ*craA* (VCA0849), Tn*7::gfp,* Sm^r^ Gm^r^ | This study |
| FY_Vc_12179 | FY_Vc_514 Δ*lapG* (VCA1081), Tn*7::gfp,* Sm^r^ Gm^r^ | This study |
| FY_Vc_12177 | FY_Vc_514 Δ*lapD* (VCA1082-83), Tn*7::gfp,* Sm^r^ Gm^r^ | This study |
| **Plasmids** |  |  |
| pBBRlux | *luxCDABE*-based promoter fusion vector, Cm^r^ | (9) |
| pFY_1284 | pBBRlux *frhA* (VC1620) promoter, Cm^r^ (-397, +9) | This study |
| pFY_1286 | pBBRlux *craA* (VCA0849) promoter, Cm^r^ (-300, +9) | This study |
| pGP704-*sacB*28 | pGP704 derivative; *mob-oriT sacB*, Ap^r^ | G. Schoolnik |
| pFY_1482 | pGP704-*sacB*28::Δ*lapG*, Ap^r^ | This study |
| pFY_1485 | pGP704-*sacB*28::Δ*lapD*, Ap^r^ | This study |
| pFY_318 | pGP704-*sacB*28::Δ*frhA*, Ap^r^ | This study |
| pFY_296 | pGP704-*sacB*28::Δ*craA*, Ap^r^ | This study |
| pFY_1070 | pGP704-*sacB*28::Δ*flrA*, Ap^r^ | This study |
| pFY4535 | pMMB67EH Gm resistant derivative containing the c-di-GMP dependent biosensor | (10) |
| pMCM11 | pGP704::mTn7*-gfp*, Gm^r^ Ap^r^ | M. Miller and G. Schoolnik |
| pUX-BF13 | oriR6K helper plasmid, *mob-oriT*, provides the Tn7 transposition function in *trans*, Ap^r^ | (11) |

1. Herrero M, de Lorenzo V, Timmis KN. 1990. Transposon vectors containing non-antibiotic resistance selection markers for cloning and stable chromosomal insertion of foreign genes in gram-negative bacteria. J Bacteriol 172:6557-67.

2. Simon R, Priefer U, Puhler A. 1983. A broad host range mobilization system for invivo genetic-engineering - transposon mutagenesis in gram-negative bacteria. Bio-Technology 1:784-791.

3. Yildiz FH, Schoolnik GK. 1999. *Vibrio cholerae* O1 El Tor: identification of a gene cluster required for the rugose colony type, exopolysaccharide production, chlorine resistance, and biofilm formation. Proc Natl Acad Sci U S A 96:4028-33.

4. Camilli A, Mekalanos JJ. 1995. Use of recombinase gene fusions to identify *Vibrio cholerae* genes induced during infection. Mol Microbiol 18:671-83.

5. Yildiz FH, Dolganov NA, Schoolnik GK. 2001. VpsR, a member of the response regulators of the two-component regulatory systems, is required for expression of *vps* biosynthesis genes and EPS(ETr)-associated phenotypes in *Vibrio cholerae* O1 El Tor. J Bacteriol 183:1716-26.

6. Jones CJ, Utada A, Davis KR, Thongsomboon W, Zamorano Sanchez D, Banakar V, Cegelski L, Wong GC, Yildiz FH. 2015. C-di-GMP regulates motile to sessile transition by modulating MshA pili biogenesis and near-surface motility behavior in *Vibrio cholerae*. PLoS Pathog 11:e1005068.

7. Beyhan S, Tischler AD, Camilli A, Yildiz FH. 2006. Transcriptome and phenotypic responses of *Vibrio cholerae* to increased cyclic di-GMP level. J Bacteriol 188:3600-13.

8. Casper-Lindley C, Yildiz FH. 2004. VpsT is a transcriptional regulator required for expression of *vps* biosynthesis genes and the development of rugose colonial morphology in *Vibrio cholerae* O1 El Tor. J Bacteriol 186:1574-8.

9. Lenz DH, Mok KC, Lilley BN, Kulkarni RV, Wingreen NS, Bassler BL. 2004. The small RNA chaperone Hfq and multiple small RNAs control quorum sensing in *Vibrio harveyi* and *Vibrio cholerae*. Cell 118:69-82.

10. Zamorano-Sanchez D, Xian W, Lee CK, Salinas M, Thongsomboon W, Cegelski L, Wong GCL, Yildiz FH. 2019. Functional specialization in *Vibrio cholerae* diguanylate cyclases: distinct modes of motility suppression and c-di-GMP production. MBio 10: e00670-19.

11. Bao Y, Lies DP, Fu H, Roberts GP. 1991. An improved Tn7-based system for the single-copy insertion of cloned genes into chromosomes of gram-negative bacteria. Gene 109:167-8.
