# Supplementary material for: A Conserved Regulatory Circuit Controls Large Adhesins in Vibrio cholerae
Source: mBio. 2019 Dec 3;10(6):e02822-19. doi: 10.1128/mBio.02822-19 (PMC6890996; doi:10.1128/mBio.02822-19)
Supplement: Supplementary file 5 [file mBio.02822-19-sf002.docx]

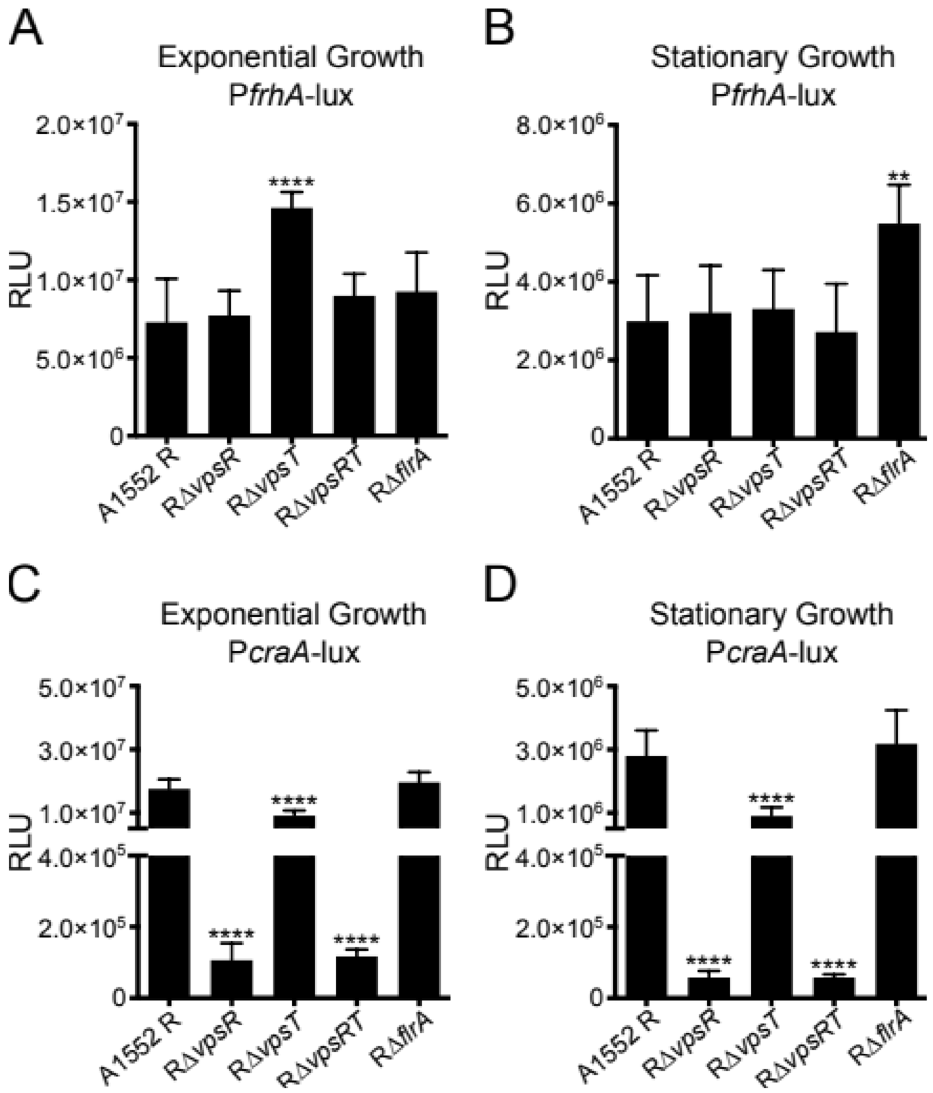

A
Exponential Growth
PfrhA-lux
RLU
2.0×10^7
1.5×10^7
1.0×10^7
5.0×10^6
0
****
A1552 R
RΔvpsR
RΔvpsT
RΔvpsRT
RΔflrA
B
Stationary Growth
PfrhA-lux
RLU
8.0×10^6
6.0×10^6
4.0×10^6
2.0×10^6
0
**
A1552 R
RΔvpsR
RΔvpsT
RΔvpsRT
RΔflrA
C
Exponential Growth
PcraA-lux
RLU
5.0×10^7
3.0×10^7
1.0×10^7
4.0×10^5
2.0×10^5
0
****
****
****
A1552 R
RΔvpsR
RΔvpsT
RΔvpsRT
RΔflrA
D
Stationary Growth
PcraA-lux
RLU
5.0×10^6
3.0×10^6
1.0×10^6
4.0×10^5
2.0×10^5
0
****
****
****
A1552 R
RΔvpsR
RΔvpsT
RΔvpsRT
RΔflrA
